# A New Baurusuchid (Crocodyliformes, Mesoeucrocodylia) from the Late Cretaceous of Brazil and the Phylogeny of Baurusuchidae

**DOI:** 10.1371/journal.pone.0021916

**Published:** 2011-07-13

**Authors:** Felipe C. Montefeltro, Hans C. E. Larsson, Max C. Langer

**Affiliations:** 1 Departamento de Biologia, Faculdade de Filosofia, Ciências e Letras de Ribeirão Preto – Universidade de São Paulo, Ribeirão Preto, Brazil; 2 Redpath Museum, McGill University, Montréal, Canada; Raymond M. Alf Museum of Paleontology, United States of America

## Abstract

**Background:**

Baurusuchidae is a group of extinct Crocodyliformes with peculiar, dog-faced skulls, hypertrophied canines, and terrestrial, cursorial limb morphologies. Their importance for crocodyliform evolution and biogeography is widely recognized, and many new taxa have been recently described. In most phylogenetic analyses of Mesoeucrocodylia, the entire clade is represented only by *Baurusuchus pachecoi*, and no work has attempted to study the internal relationships of the group or diagnose the clade and its members.

**Methodology/Principal Findings:**

Based on a nearly complete skull and a referred partial skull and lower jaw, we describe a new baurusuchid from the Vale do Rio do Peixe Formation (Bauru Group), Late Cretaceous of Brazil. The taxon is diagnosed by a suite of characters that include: four maxillary teeth, supratemporal fenestra with equally developed medial and anterior rims, four laterally visible quadrate fenestrae, lateral Eustachian foramina larger than medial Eustachian foramen, deep depression on the dorsal surface of pterygoid wing. The new taxon was compared to all other baurusuchids and their internal relationships were examined based on the maximum parsimony analysis of a discrete morphological data matrix.

**Conclusion:**

The monophyly of Baurusuchidae is supported by a large number of unique characters implying an equally large morphological gap between the clade and its immediate outgroups. A complex phylogeny of baurusuchids was recovered. The internal branch pattern suggests two main lineages, one with a relatively broad geographical range between Argentina and Brazil (Pissarrachampsinae), which includes the new taxon, and an endemic clade of the Bauru Group in Brazil (Baurusuchinae).

## Introduction

Baurusuchid crocodyliforms have been long recognized by their highly divergent morphology relative to other crocodyliforms. The good preservation of the holotype skull of *Baurusuchus pachecoi* [1] made the taxon present in nearly every phylogenetic analysis of Crocodyliformes (**e. g.:** [2]–[14]). As a consequence, this species plays a central role in interpreting basal crocodyliform evolutionary and biogeographic patterns [2], [15]–[17]. On the other hand, a comprehensive phylogenetic analysis of putative baurusuchids has never been done, and the current and only phylogenetic definition for the clade was not backed up by a phylogenetic analysis [18]. Consequently, baurusuchid membership and internal relationships are not well studied or resolved [19]. Previously assigned taxa from the Late Cretaceous of South America [18], [20]–[23] are commonly accepted as members of the group, but this is more uncertain for fragmentary taxa from Cretaceous deposits of Argentina and Pakistan and Paleogene deposits of Europe and Africa [17], [18], [24]–[27]. The baurusuchid affinity of these forms is debated, and many current analyses do not support this association [9], [19], [28]–[31]. *Baurusuchus* is certainly a key taxon in crocodyliform phylogeny, but its use as the only baurusuchid in phylogenetic analyses (**e. g.:** [9], [13], [32]–[34]) underestimates the morphological diversity of the clade and may influence results because of its derived characters.

**Figure 1:**
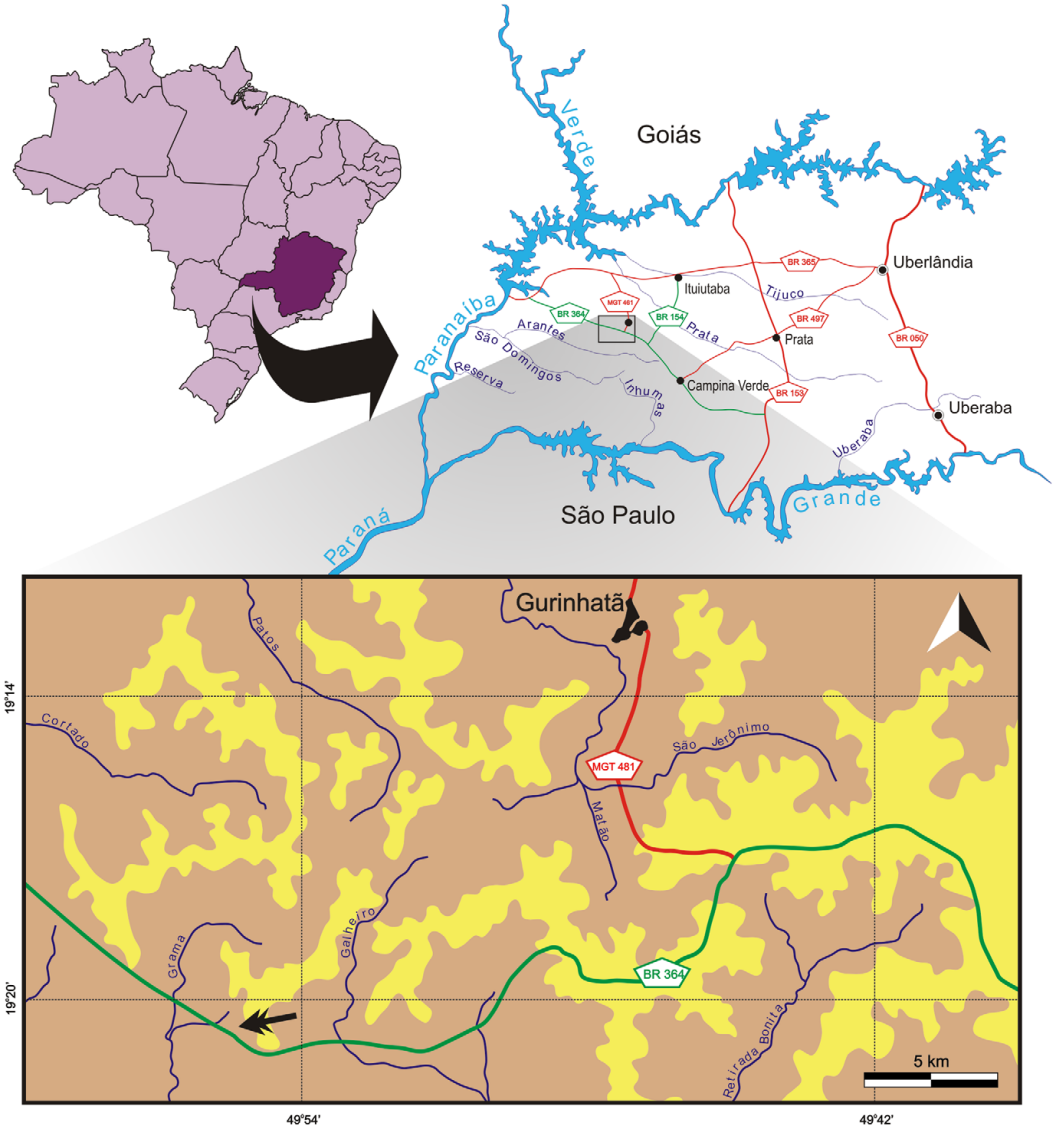
Map of the area where *Pissarrachampsa sera* was collected. Area highlighted in maps of Brazil, Minas Gerais (above on the left), and the Triângulo Mineiro region (above on the right). Main river courses indicated in blue, paved roads in red, and secondary non-paved roads in green. Surface distribution of the Bauru Group rocks based on [106]: beige  =  Vale do Rio do Peixe Formation; yellow  =  Marília Formation.

**Figure 2:**
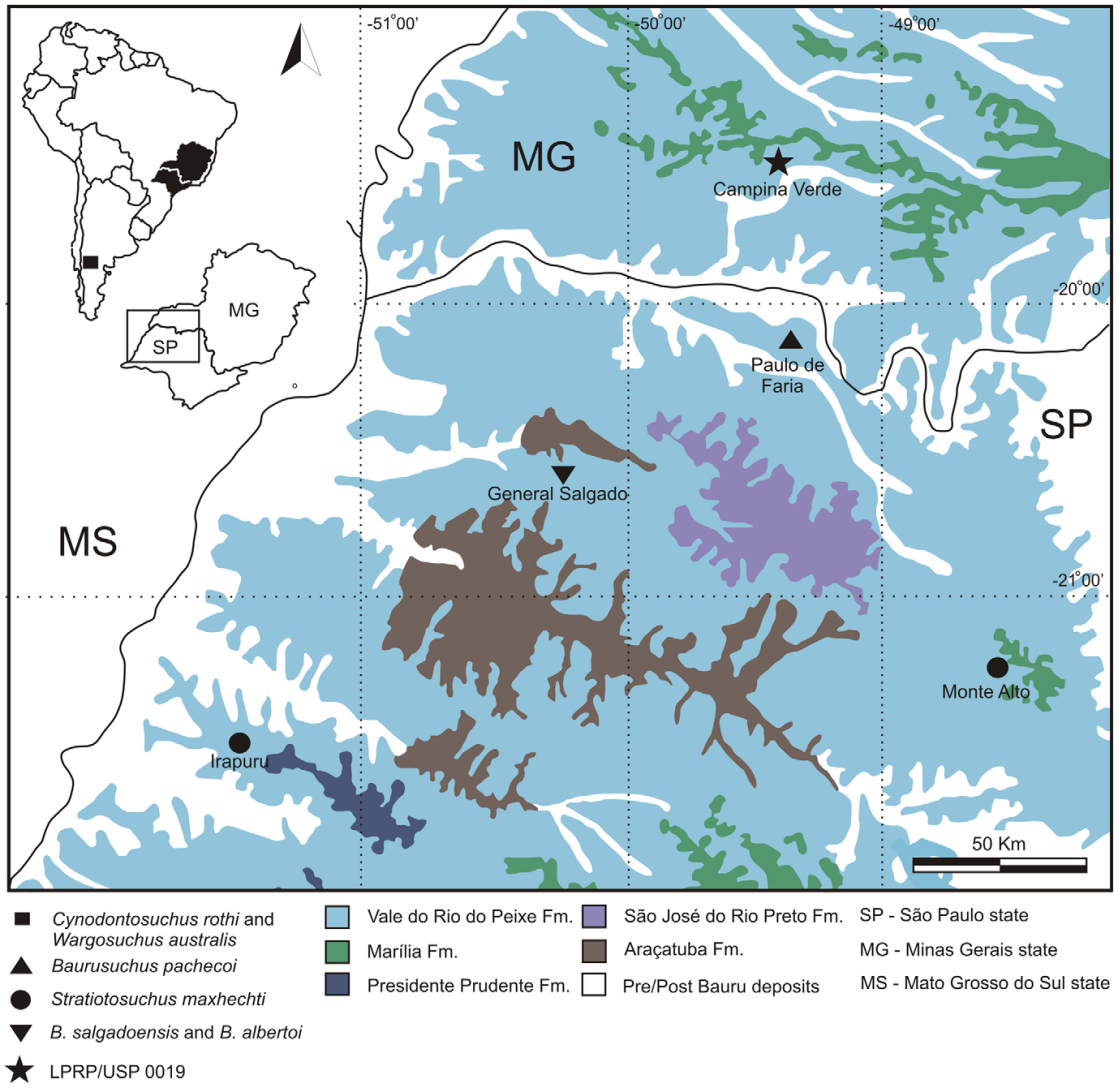
Surface distribution of the Bauru Group rocks. The northeastern portion of the Bauru Basin (São Paulo and Minas Gerais states), depicting the localities where Baurusuchidae fossils were collected (modified from [107]).

Here we describe a new baurusuchid based on a well preserved skull and a referred partial skull and lower jaw. The specimens are accessioned in the collection of the Laboratório de Paleontologia de Ribeirão Preto (LPRP), Faculadade de Filosofia Ciências e Letras de Ribeirão Preto (FFCLRP), Universidade de São Paulo (USP) as LPRP/USP 0018 and LPRP/USP 0019, and come from the municipality of Campina Verde, in the region known as Triângulo Mineiro, Minas Gerais state, Brazil (Figure 1). The material was collected in June 2008 during ongoing systematic field work by members of the Laboratório de Paleontologia de Ribeirão Preto, FFCLRP-USP, from deposits of the Vale do Rio do Peixe Formation (Figure 2), which is composed of fine-grained sandstones with sandy-mudstone contributions deposited under a continental, semi-arid climate [35], [36].

The new taxon is undoubtedly a baurusuchid, based on its derived dog-like skull and hypertrophied canines. We tested the taxon's phylogenetic position with the first comprehensive analysis of the better known putative bauruschids. Characters in the phylogenetic analysis were compiled from previous studies, and a suite of new characters were added to account for previously unrecognized transformation series. The analysis clarifies the neglected internal relationships of Baurusuchidae and presents a set of new apomorphies for the clade. We also present a set of formal definitions for the group.

## Methods

### Preparation

The fossil material was mechanically prepared using pin vice and pneumatic air scribe. Paraloid B72 dissolved in acetone was used for surface consolidation and as an adhesive.

### Nomenclatural Acts

The electronic version of this document does not represent a published work according to the International Code of Zoological Nomenclature (ICZN), and hence the nomenclatural acts contained in the electronic version are not available under that Code from the electronic edition. Therefore, a separate edition of this document was produced by a method that assures numerous identical and durable copies, and those copies were simultaneously obtainable (from the publication date noted on the first page of this article) for the purpose of providing a public and permanent scientific record, in accordance with Article 8.1 of the Code. The separate print-only edition is available on request from PLoS by sending a request to PLoS ONE, 185 Berry Street, Suite 3100, San Francisco, CA 94107, USA along with a check for $10 (to cover printing and postage) payable to “Public Library of Science”. In addition, this published work and the nomenclatural acts it contains have been registered in ZooBank, the proposed online registration system for the ICZN. The ZooBank LSIDs (Life Science Identifiers) can be resolved and the associated information viewed through any standard web browser by appending the LSID to the prefix “http://zoobank.org/”. The LSID for this publicaftion is: urn:lsid:zoobank.org:pub:023DCB79-D033-4FB0-81C0-28302E440E15.

### Ethics statement

No live animals were used in this study.

### Field work permission

The permission for fossil collection was obtained from Deparftamento Nacional de Produção Mineral (document: 36/DIFIS) based on ordinance n° 4.146 from 4^th^ March, 1942.

### Systematic paleontology

#### Crocodyliformes

Benton & Clark 1988 [2]

#### Mesoeucrocodylia

Whetstone & Whybrown 1983 [37] *sensu* Benton & Clark 1988 [2]

#### Baurusuchidae

Price, 1945 [1]

***Pissarrachampsa* gen. nov.**

#### Derivation of name

The generic epithet is a combination of the local name for the fossil bearing sandstones, piçarra, and the Greek suffix Χάμψαι (latinized as “champsa”) meaning crocodile.

#### Type species

*Pissarrachampsa sera*

urn:lsid:zoobank.org:act:F69BDAD1-38B7-4F89-9591-2C503826C91E

#### Diagnosis

Same as for the only known species.

*Pissarrachampsa sera* **gen. et. sp. nov.** (Figures 3, 4, 5, 6, 7, 8, 9, 10, 11, 12, 13, 14, 15 and 16)

**Figure 3:**
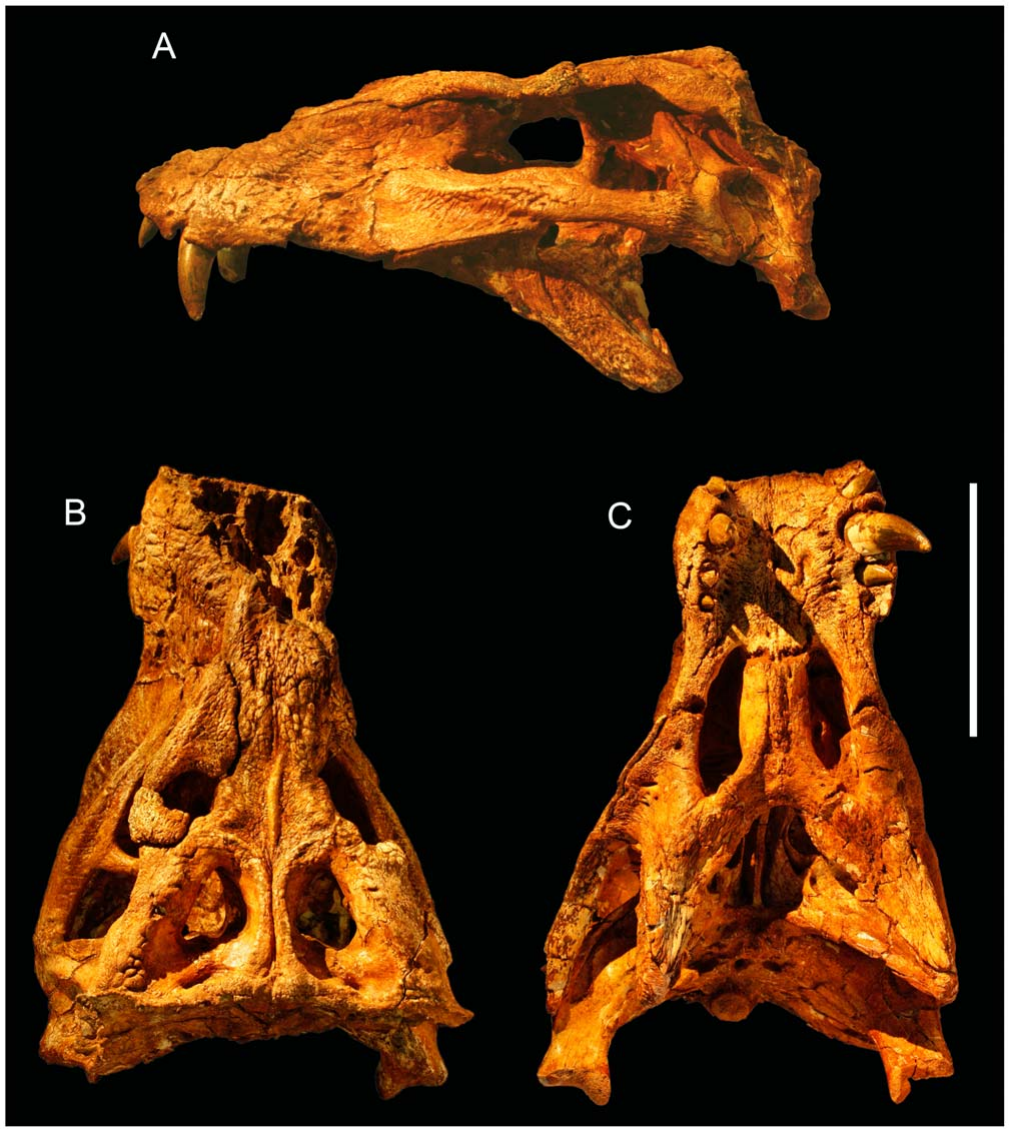
Skull of the baurusuchid *Pissarrachampsa sera* (LPRP/USP 0019, holotype). A) lateral; B) dorsal; C) ventral views. B and C have depth of field calibrated to the skull table and palatal plane respectively. Scale bar equals 10 cm.

**Figure 4:**
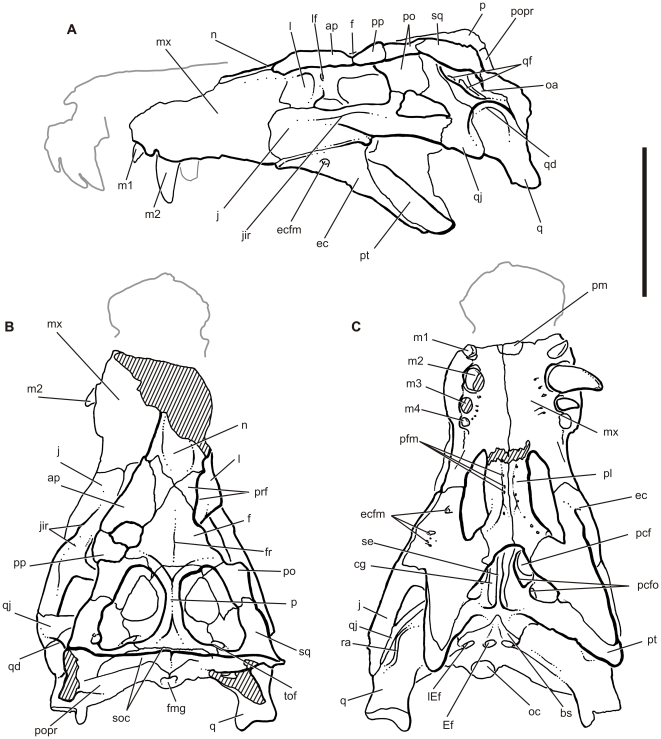
Skull of the baurusuchid *Pissarrachampsa sera*. A) and C) Drawing of the skull LPRP/USP 0019 (holotype) in strict lateral (left) and ventral views, respectively. B) Reconstruction of the dorsal surface of the same skull, based on a strict dorsal view of skull roof and a slightly posterodorsal view of the occipital plane. Hatched areas indicate broken surfaces. Reconstruction of the anterior part of the rostrum based on the referred specimen (LPRP/USP 0018). Abbreviations: ap, anterior palpebral; bs, basisphenoid; cg, choanal groove; ec, ectopterygoid; ecfm, ectopterygoid foramina; Ef, medial Eustachian foramen; f, frontal; fmg, foramen magnum; fr, frontal ridge; j, jugal; jir, jugal infraorbital ridge; l, lacrimal; lEf, lateral Eustachian foramen; lf, lacrimal duct foramen; mx, maxilla; m1-m4, maxillary teeth (1–4); n, nasal; oa, otic aperture; oc, occipital condyle; p, parietal; pcf, parachoanal fenestra; pcfo, parachoanal fossae; pfm, palatine foramina; pfp, pl, palatine; pm, premaxilla; po, postorbital; popr, paraoccipital process; pp, posterior palpebral; prf, prefrontal; pt, pterygoid; q, quadrate; qd, quadrate depression; qf, quadrate fenestrae; qj, quadratojugal; ra, ridge ‘A’; se, choanal septum; soc, supraoccipital; sq, squamosal; tof, temporo-orbital foramen. Scale bar equals 10 cm.

**Figure 5:**
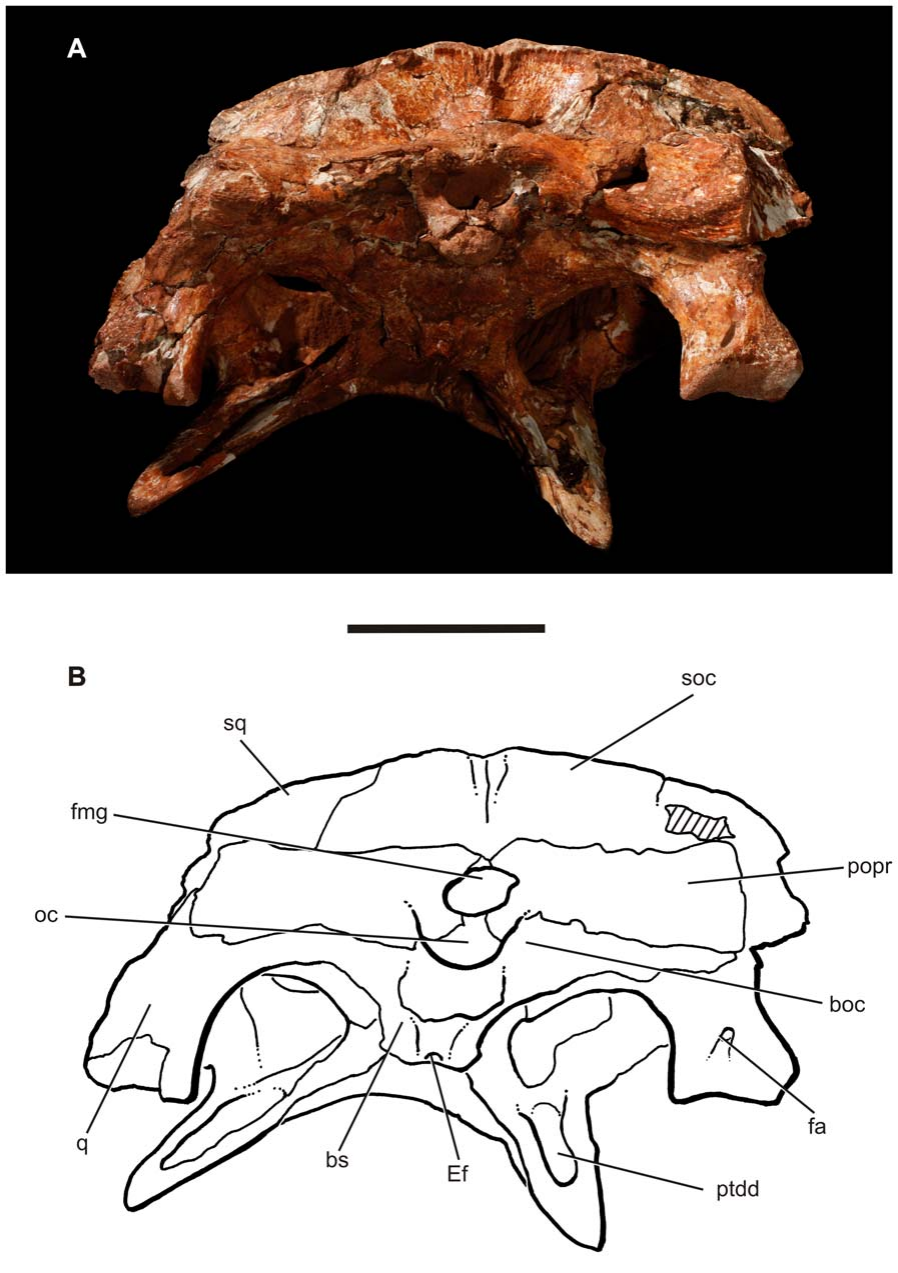
Skull of the baurusuchid *Pissarrachampsa sera* (LPRP/USP 0019, holotype). A) occipital view; B) interpretive drawing of the occipital view. Abbreviations: boc, basioccipital; fa, foramen aërum; ptdd, pterygoid dorsal depression. Scale bar equals 5 cm.

**Figure 6:**
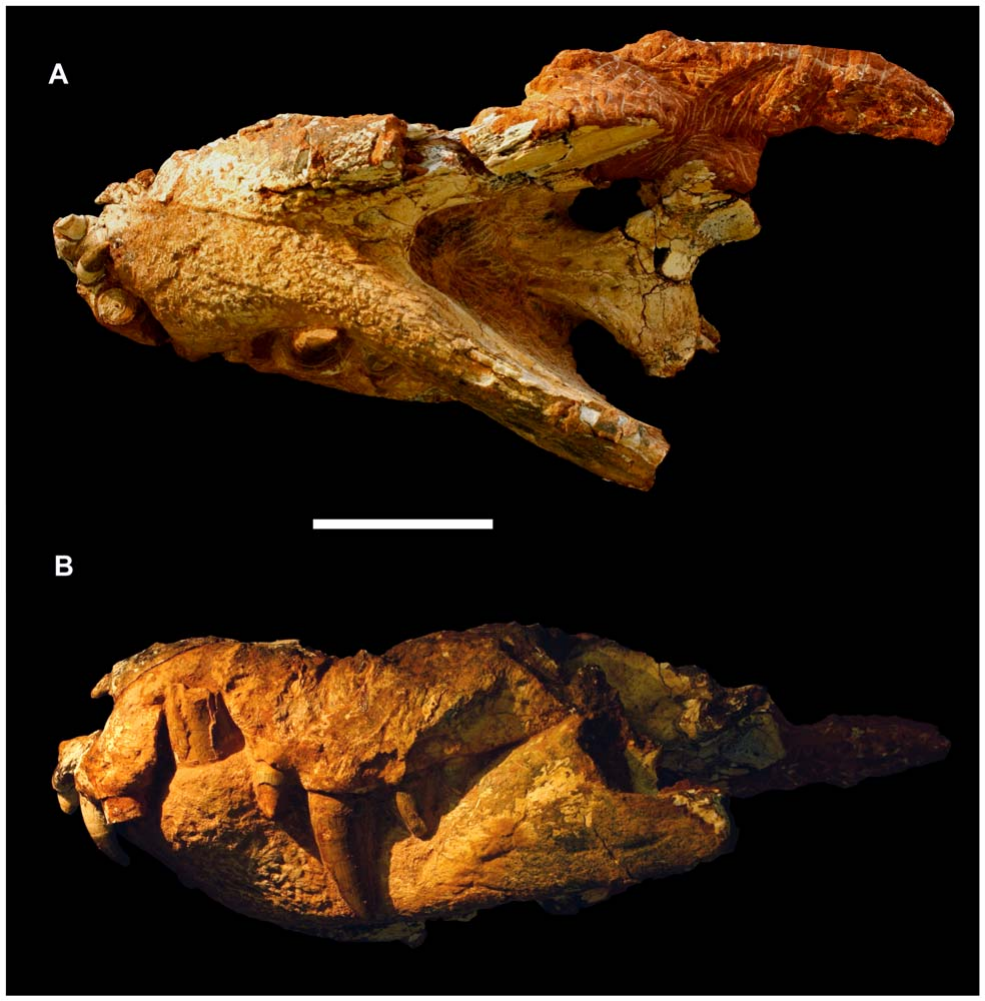
Skull of the referred specimen of *Pissarrachampsa sera* (LPRP/USP 0018). A) ventral; B) lateral (mirrored) views. Scale bar equals 5 cm.

**Figure 7:**
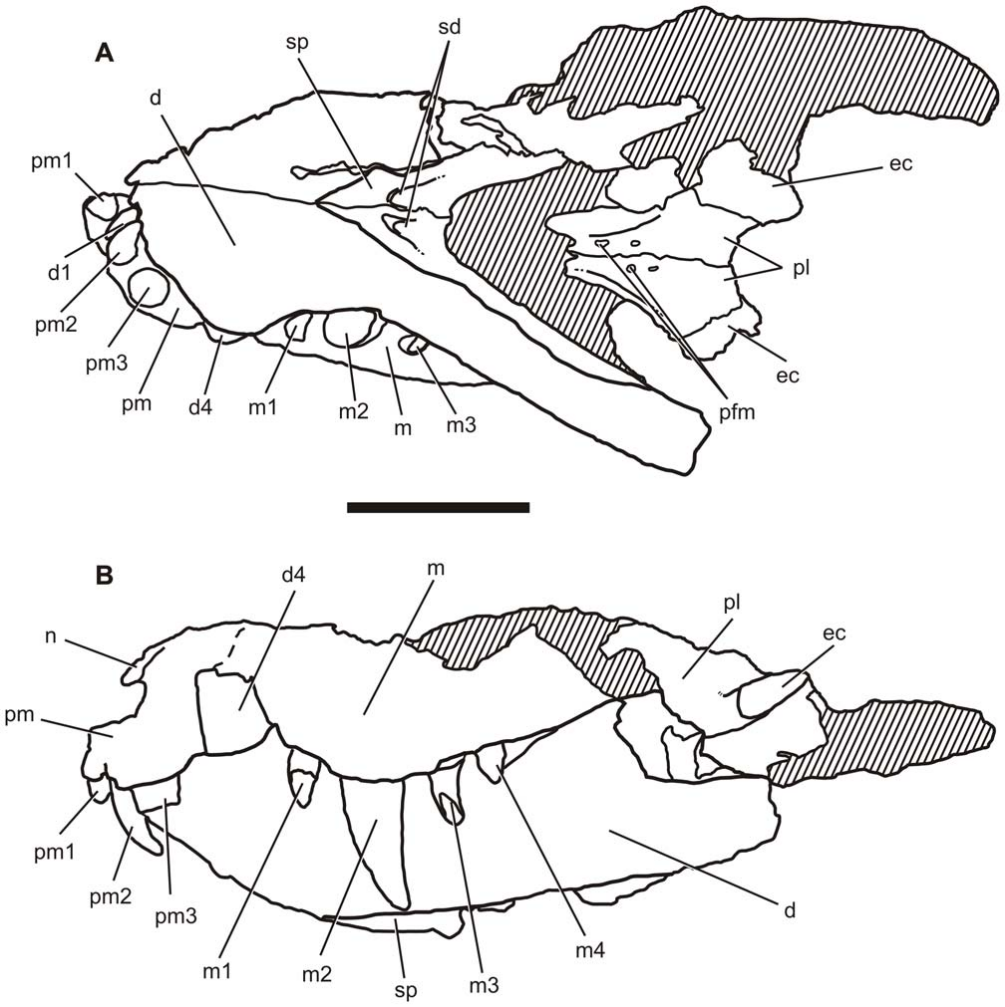
Drawing of the skull of the referred specimen of *Pissarrachampsa sera* (LPRP/USP 0018). A) ventral; B) lateral (mirrored). Dashed line represents possible premaxilla-maxilla suture. Hatched areas indicate broken surfaces and matrix. Abbreviations: d, dentary; d1, dentary tooth 1; d4, dentary tooth 4; premaxilla; pm1-pm3, premaxillary teeth 1–3; sd, splenial depressions; sp, splenial. Scale bar equals 5 cm.

**Figure 8:**
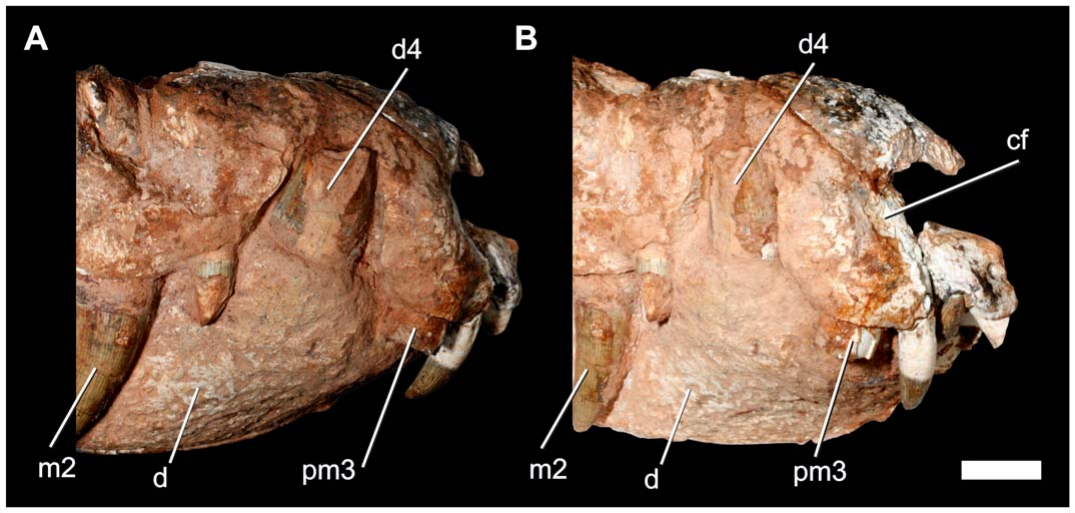
Details of the right anteriormost portion of the rostrum of *Pissarrachampsa sera* (LPRP/USP 0018). A) lateral; B) anterolateral views. Abbreviation: cf, circumnarial fossa. Scale bar equals 1 cm.

**Figure 9:**
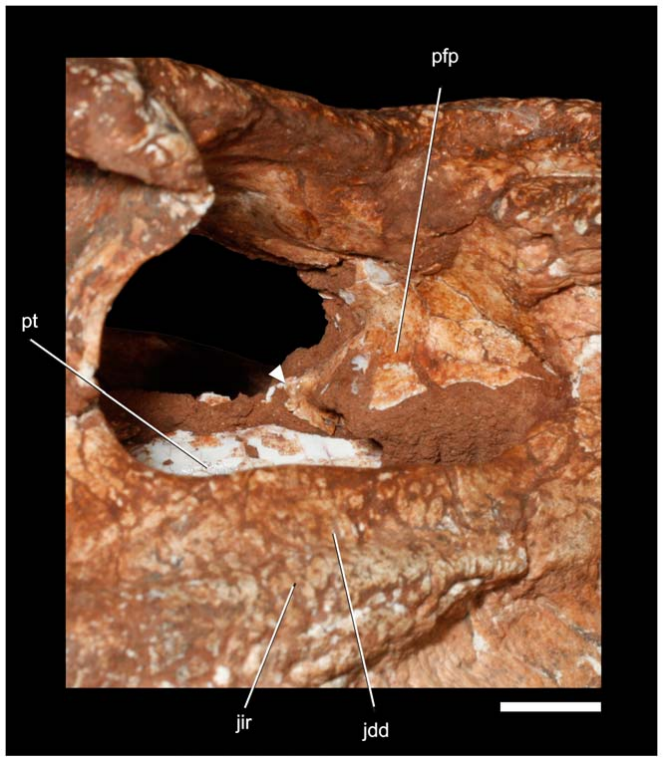
Details of the right prefrontal pillar of *Pissarrachampsa sera* (LPRP/USP 0019) through the orbit. Arrow points to the medioventral extension of prefrontal pillar. Abbreviations: jdd, jugal dorsal depression; pfp, prefrontal pillar. Scale bar equals 1 cm.

**Figure 10:**
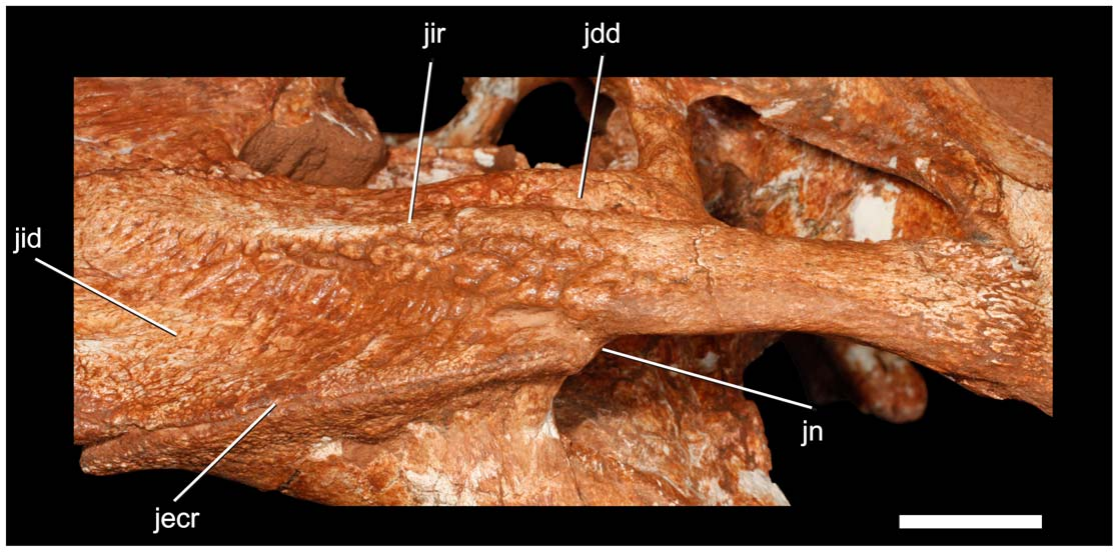
Details of the left cheek area of *Pissarrachampsa sera* (LPRP/USP 0018). Abbreviations: jecr, jugal-ectopterygoid ridged suture; jid, jugal infraorbital depression; jn, jugal notch. Scale bar equals 1 cm.

**Figure 11:**
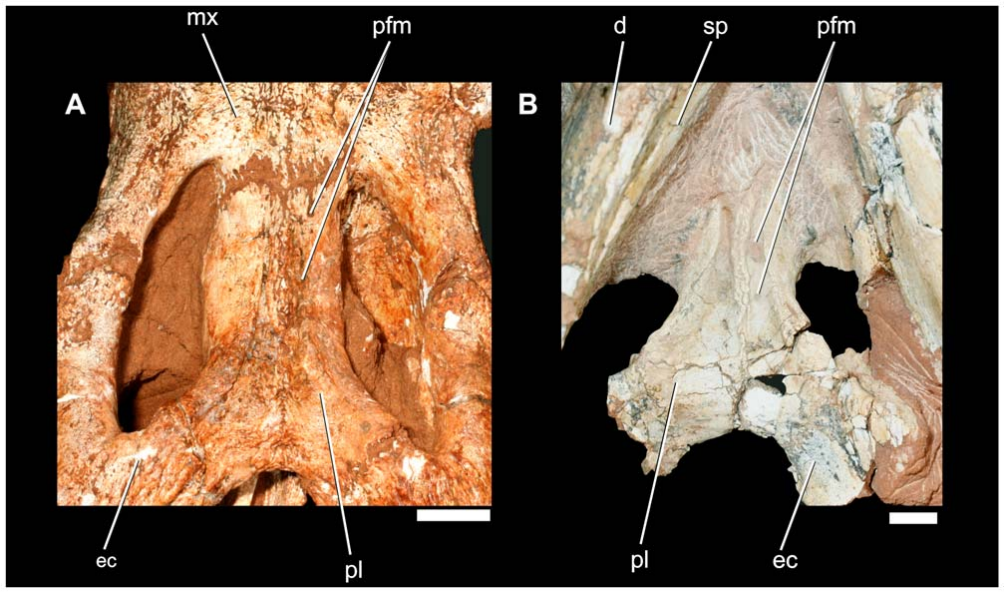
Details of the palatine ventral surface of *Pissarrachampsa sera*. A) LPRP/USP 0019; B) LPRP/USP 0018. Scale bar equals 1 cm.

**Figure 12:**
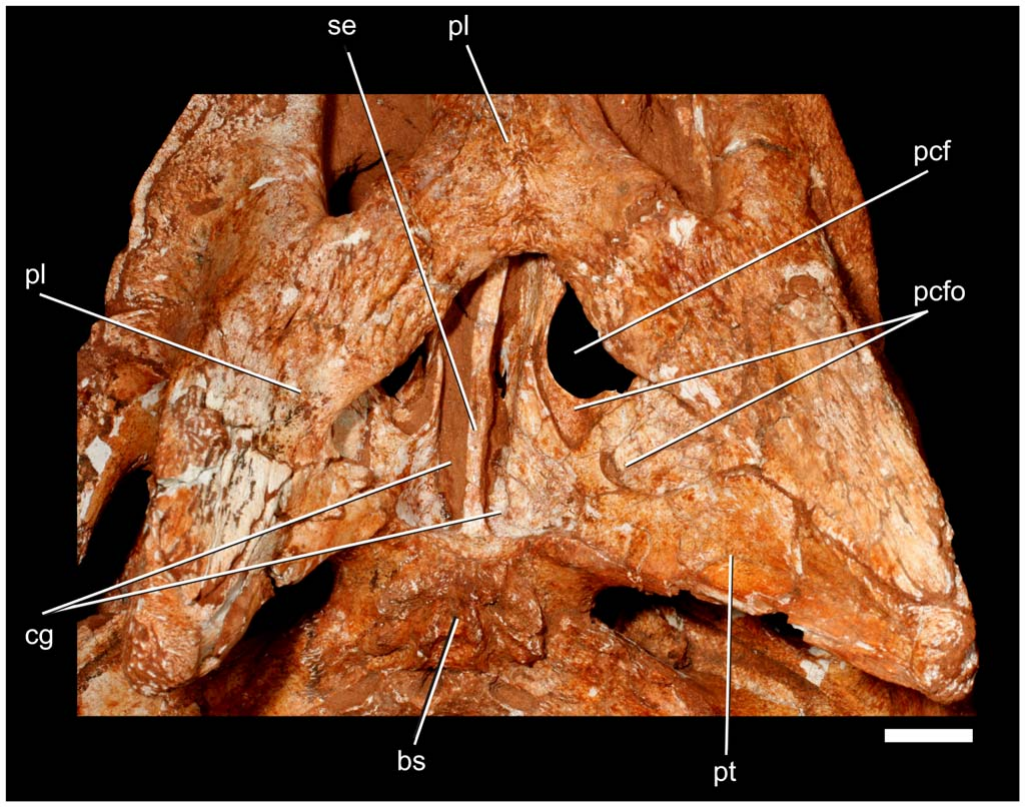
Details of the choanal region of *Pissarrachampsa sera* (LPRP/USP 0019). Scale bar equals 1 cm.

**Figure 13:**
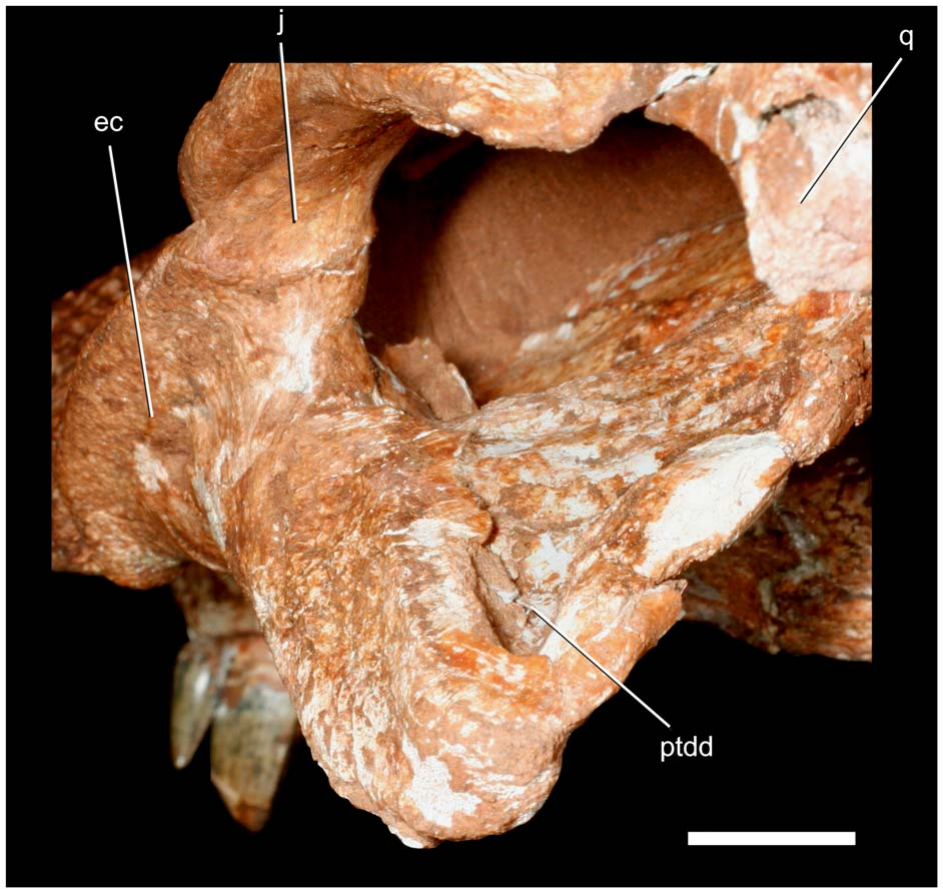
Details of the dorsal surface of the left pterygoid wing of *Pissarrachampsa sera* (LPRP/USP 0019). Image taken from posteroventral view. Scale bar equals 1 cm.

**Figure 14:**
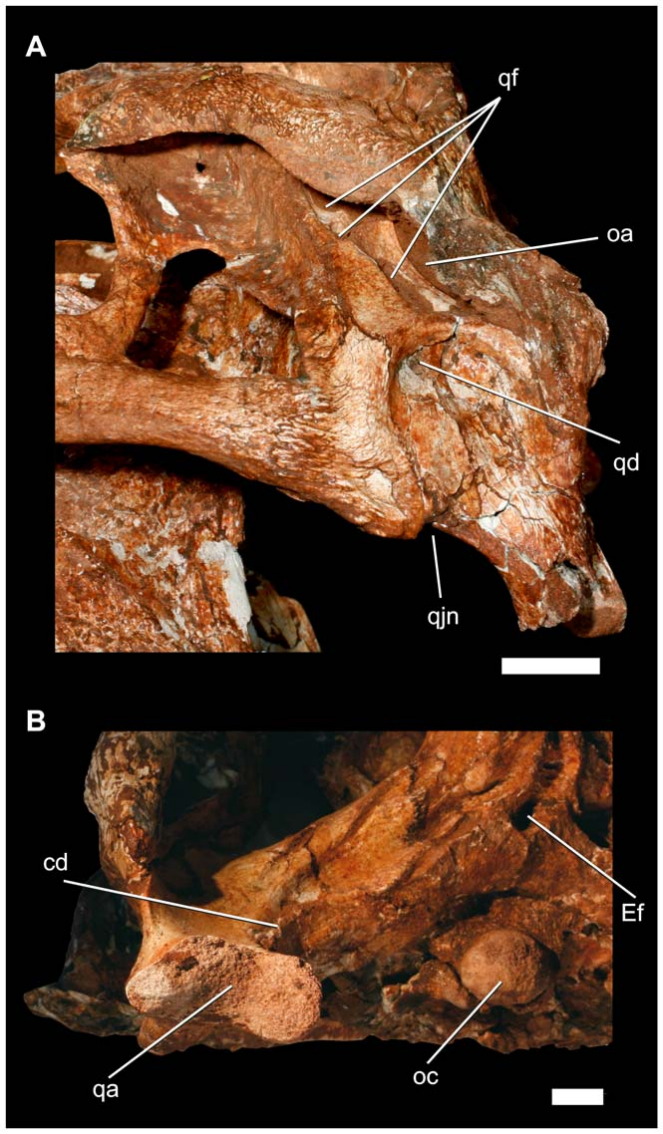
Details of the left posterolateral region of the cranium of *Pissarrachampsa sera* (LPRP/USP 0019). A) lateral view; B) ventral view. Abbreviations: qa, quadrate articulation; qjn, quadratojugal notch. Scale bars equals 1 cm.

**Figure 15:**
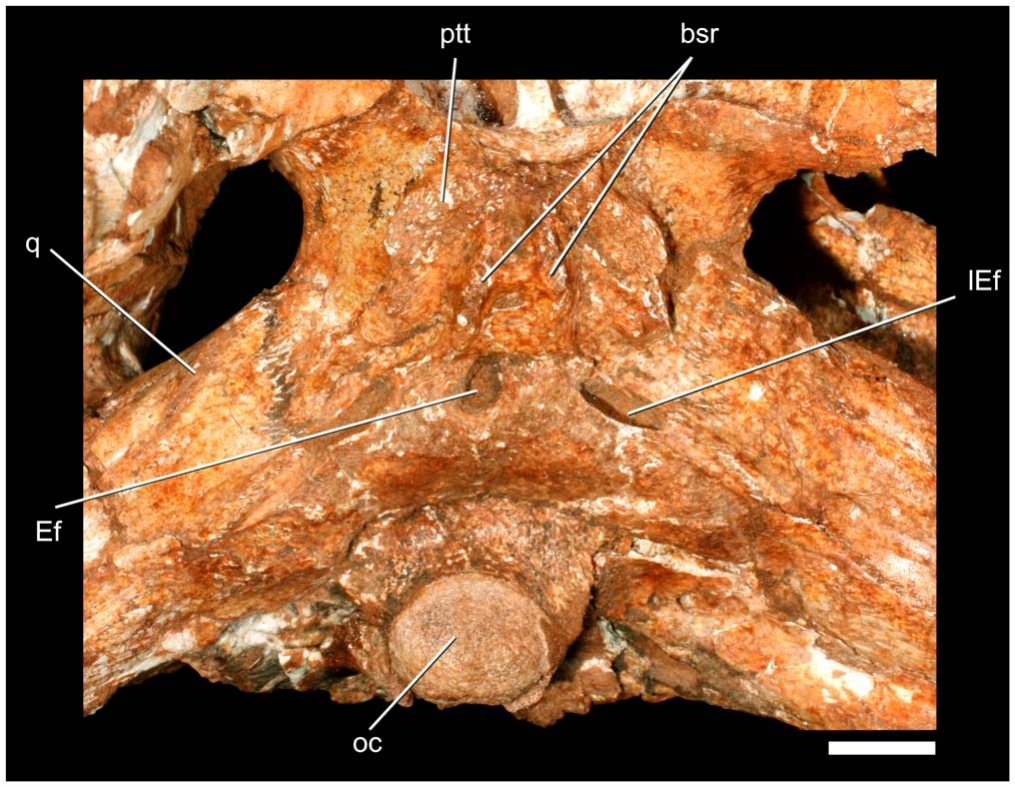
Details of the ventral portion of neurocranium of *Pissarrachampsa sera* (LPRP/USP 0019). Abbreviations: bsr, basisphenoid ridges; ptt, pterygoid tuberosity. Scale bar equals 1 cm.

**Figure 16:**
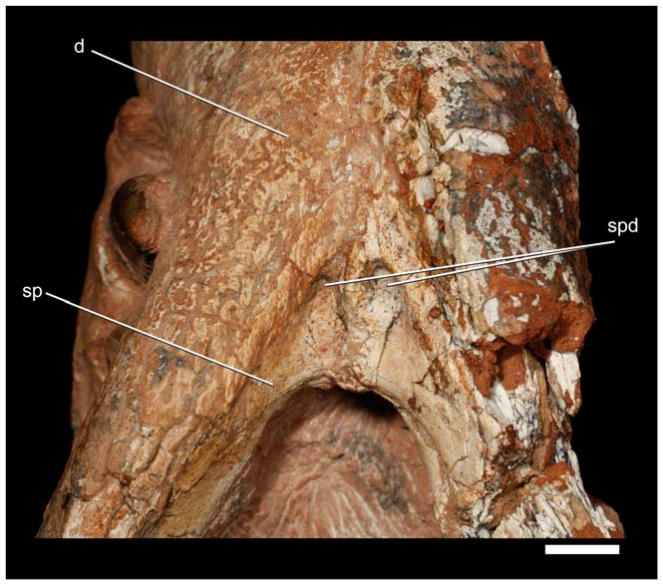
Detail of the ventral portion of symphysis of *Pissarrachampsa sera* (LPRP/USP 0018). Scale bar equals 1 cm.

urn:lsid:zoobank.org:act:AF7428F1-B88C-4212-A543-666593C75340

#### Derivation of name

The specific epithet is a Latin word meaning ‘late’, in reference to the collection of the holotype. It was the last fossils found during the 2008 expedition and were about to be left behind because of the tight schedule. In addition, sera is also a homage to the Minas Gerais state, where the fossils were found, the flag of which contains Virgil's inscription: “Libertas Quæ Sera Tamen” meaning “Freedom Albeit Late”.

#### Holotype

**LPRP/USP 0019** is a nearly complete cranium, lacking only the anteriormost portion of the rostrum, right palpebrals, and mandibles.

#### Referred specimen

**LPRP/USP 0018** is a partial rostrum and anterior palate with the anterior portion of the mandibles articulated.

#### Type locality

Inhaúmas-Arantes Farm, Campina Verde municipality, Minas Gerais, Brazil (19°20′ 41.8″ S; 49°55′ 12,9″ W).

#### Age and horizon

Vale do Rio do Peixe Formation, Bauru Group, Bauru Basin; Late Cretaceous (Turonian-Santonian or Campanian-Maastrichtian, but see discussion).

#### Diagnosis

Baurusuchid with four maxillary teeth, a longitudinal depression on the anterior portion of frontal, frontal longitudinal ridge extending anteriorly overcoming the frontal midlength, supratemporal fenestra with equally developed medial and anterior rims, lacrimal duct at the corner formed by the dorsal (support for anterior palpebral) and lateral lacrimal surfaces, well developed rounded foramen between the palpebrals, quadratojugal and jugal do not form a continuous ventral border (a notch is present due to the ventral displacement of the quadratojugal), four quadrate fenestrae visible laterally, quadrate lateral depression with anteroposteriorly directed major axis, sigmoidal muscle scar in the medial surface of the quadrate (ridge ‘A’), ectopterygoid almost reaching the posterior margin of the pterygoid wings, a single ventral parachoanal fenestra, and one ventral parachoanal fossa (divided into medial and lateral parachoanal subfossae), lateral Eustachian foramina larger than the medial one, and a deep depression on the posterodorsal surface of the pterygoid wings.

### Description

The anatomical description and soft tissue inferences will follow [9], [38]–[44] as much as possible. The dental description follows the definitions and nomenclature of [45], [46]. The supratemporal morphology of baurusuchids differs from many crocodyliforms in that the dorsal and ventral portions of the supratemporal chamber are demarcated by a distinct ridge. We will follow the argumentation of [47], regarding the antorbital fenestra, and describe the dorsal and ventral portions of the supratemporal chamber as external and internal, respectively. The dorsal surfaces of the bones surrounding the supratemporal fenestrae are often excavated into a depression usually termed the supratemporal fossa, which is also used here. The dorsal peripheral rim of the fossa marks the external supratemporal fenestra and roughly corresponds to the “supratemporal fenestra” as traditionally used. The bony edge ventral to the supratemporal fossa is the internal supratemporal fenestra. The internal and external supratemporal fenestrae are hard to distinguish in Crocodyliformes with a poorly developed supratemporal fossa; i.e., in some eusuchians there is only a single fenestra. This becomes more complicated because of the verticalization of the supratemporal fossa, the unequally developed regions of the fossa, and the confluence of the supratemporal fossa with the lateral surface of the orbitotemporal bones in some groups. However, for *Pissarrachampsa sera* and other baurusuchids, the separation between external and internal supratemporal fenestrae is useful.

### Cranium

The following anatomical description is primarily based on the cranium LPRP/USP 0019 (Figures 3,4,9, 10, 11, 12, 13, 14, 15). LPRP/USP 0018 (Figures 6, 7, 8, 16) is also employed in the description of the anterior portion of rostrum, mandible and dentition, and the minor morphological differences on their overlapping elements are discussed when they occur. The more evident preservational deficiencies of the holotype specimen are the lack of the anterior portion of the rostrum due to erosion, the loss of the right palpebral bones, and fractures on the occipital region and quadrate condyles. Some minor damages and breakages in the skull roof, braincase, and palatal region are also present. As a result of pressure inflicted during fossilization, the sides of the snout and the pterygoid wings are no longer symmetrically aligned, so that the skull appears twisted towards the right when viewed from the front and from behind. Also as an effect of such distortion, the right postorbital bar is broken at the level of the postorbital-jugal contact, and its dorsal tip pierces the postorbital descending flange.

In dorsal view, LPRP/USP 0019 has a triangular shape. The combined morphologies of LPRP/USP 0018 and 0019 allow the reconstruction of a relatively short snout (Table 1-2, Figure 3,4,6,7). The maximum length of the preserved rostrum of LPRP/USP 0019 is about 42% of the basal skull length. The complete preorbital length is estimated to be approximately 70% of the entire skull length, placing *Pissarrachampsa* in the category of short snouted Crocodyliformes [38]. The snout is not conspicuously constricted in relation to the postorbital portion of the skull, and the skull table is continuous with the dorsal surface of the snout. The external surfaces of most of the superficial dermal bones are sculptured with irregular pits and ridges. This sculpturing is pronounced on the prefrontal, anterior portion of the frontal, postorbital dorsal surface, jugal, and squamosal and is completely absent on the supratemporal fossa, postorbital descending flange, and all but the ventral portion of the quadratojugal.

**Table 1:** Selected measurements (in cm) for the skull LPRP/USP 0019.

LPRP/USP 0019	
Preserved basal skull length (from tip of snout to occipital condyle along midline)	26.36
Preserved length of skull (from posterior end of skull table to tip of snout, on midline)	25.06
Preserved length of snout (from anterior end of orbit to tip of snout)	11.31
Greatest transverse width of skull (across quadratojugals)	17.22
Least transverse interorbital distance	3.6
Transverse width of skull at level of anterior ends of orbits	4.78
Transverse width of skull at level of postorbital bars	10.5
Transverse width of skull table anteriorly	6.73
Transverse width of skull table posteriorly	15.17

**Table 2:** Selected measurements (in cm) for the skull LPRP/USP 0018.

LPRP/USP 0018	
Preserved length of skull (from posterior end of preserved palatines to anterior tip of snout along midline)	20.28
Preserved length of snout (from posterior end of preserved maxilla to anterior tip of snout along midline)	14.52

The external nares are partially preserved in LPRP/USP 0018. They face anteriorly and were probably completely divided. The antorbital fenestra is completely closed without even a trace of the antorbital fossa. The orbits are subcircular and face anterolaterally. The anterior and posterior portions of the orbits are roofed by thick palpebrals. The external supratemporal fenestra is nearly equal in size to the orbit, and the internal supratemporal fenestra is only about half of the orbit size. The supratemporal fenestrae are longer than wide and triangular in dorsal aspect with a wide posterior margin. The triangular infratemporal fenestra is smaller than the orbit and the external supratemporal fenestra, but larger than the internal supratemporal fenestra. The infratemporal fenestra real orientation is hard to determine given the distortion of the skull. On the right side, the infratemporal fenestra faces laterally, but faces dorsolaterally on the left side. In both sides, it faces slight anteriorly. The suborbital fenestra is large, and its long axis is oriented anteroposteriorly. The choanae are wide and, together with the parachoanal structures, occupy a great part of the posterior palate. The choanae face ventrally and are limited anteriorly and anterolaterally by the palatines and ectopterygoid, and posteriorly by the pterygoid. The choanae are lozenge shaped, with the greatest axis oriented transverse of skull major axis. The anteroposterior axis is divided by a median bony septum formed by the pterygoids. The occipital region is partly damaged in LPRP/USP 0019, but a post-temporal fenestra is not present.

Only a small portion of the paired premaxillae is present as a round suture just posterior to the preserved anteroventral limit of LPRP/USP 0019. A nearly complete right premaxilla is preserved in LPRP/USP 0018. The premaxilla bounds the anteroventral, ventral, posterior, and posterodorsal edges of external nares. The nares are surrounded by a broad circumnarial fossa that faces anteriorly on its posterior portion and is not visible in lateral view (Figure 8). A short, but damaged, median protuberance is present in the ventral narial margin, suggesting that a slight infranarial prong may have contributed to the internarial bar. An obvious, small vascular foramen occurs at the lateral base of this median protuberance, and no other foramina are visible in the circumnarial fossa. The dorsal limit of the premaxilla arches over the external nares as a robust process and likely supported the lateral base of an internarial bar. The medial surface of this process preserves a fluted edge that would have contacted the right lateral edge of the nasal.

A large, laterally facing notch is present at the lateral premaxilla-maxilla suture. This notch curves ventromedially towards the palate and received an enlarged dentary caniniform tooth (d4). The notch is large enough to create a diastema between the premaxillary and maxillary dentition and a constriction in the rostrum in dorsal and lateral profiles. The exact position of the premaxilla-maxilla suture within the notch is not visible. The premaxilla has three teeth with subcircular alveolar cross-sections. The first and second are similar in size, whereas the third is hypertrophied. A deep pit is located between, and slightly distal to, the first two alveoli and probably received the first dentary tooth.

The anteroposteriorly short and dorsoventrally deep maxillae are oriented nearly vertically, comprise most of the sidewalls of the snout, and have nearly no participation in the dorsal surface of the skull. Only four teeth are housed in the maxilla. The first alveolus is relatively small and faces anteroventrally. The second alveolus has the largest anteroposterior length, with an anteroposterior length greater than the other three alveoli and opens ventrally. The third and fourth alveolus are intermediate in size and open posteroventrally. In lateral aspect, the alveolar margin is ventrally arched reaching the greatest depth at the second maxillary tooth. The short tooth row does not extent far, ending posteriorly at the level of the arched maxillary alveolar margin and well anterior to the orbits. A reduced groove with a rugose surface extends posteriorly from the tooth row. This structure may represent a remnant of the alveolar groove. An elongate groove to house the posterior teeth is common in many eusuchians. The presence of this restricted and toothless groove in *Pissarrachampsa* suggests its highly reduced tooth formula in the maxilla may be the result of loss of posterior teeth and subsequent closing of the alveolar groove during ontogeny. The lateral wall of the maxilla extends back to the level of palpebral support structures. The tooth rows are slightly arched outwards, and both ends tend to converge to their counterparts in the opposite maxilla. The internal and external alveolar walls are not symmetrical; the latter is more ventrally developed, and the lingual tooth surfaces are further exposed. Posterolaterally, the maxilla contacts the jugal and lacrimal with a short posterior projection wedged between those bones, which does not reach the orbital margin. Numerous foramina dot the palatal shelf of the maxilla just lingual to the tooth row. A deep occlusal pit for the reception of a large dentary tooth is situated immediately lingual to the alveolus of m3 and m4. An additional occlusal pit occurs on the left maxilla, medially displaced at the level of teeth 3–4 as well as small aligned deformations in palatal surface indicating a complete overbite.

Medially, the palatal shelves of the maxillae meet to form an extensive secondary bony palate. A deep longitudinal groove is present over most of the palatal shelf midline suture. This is most pronounced throughout the mid-length where three well defined foramina are also visible. The groove partially obscures the sutural contact between maxillae. Posteriorly, the groove graduates into the flat posterior maxillary palatal surface. A pair of foramina is present on the maxillary portion of the palate near the maxilla-palatine contact and appears to represent posterior extensions of the foramina of the midline groove. The palatal maxilla-palatine suture extends transversely from the midline to the medial borders of the suborbital fenestrae. Each maxilla posseses a lateral palatal ramus which extends posteriorly and abuts the ectopterygoid at the level of the midpoint of the suborbital fenestra, causing the maxilla to border not only the anterior portion of suborbital fenestra, but also its anterolateral half.

The jugal is long and transversely narrow. Its anterior (infraorbital) process expands greatly from the midlength until the contact with the maxilla and lacrimal. The orbital margin of the jugal posseses a low border that extends slightly lateral to the anterior margin of the postorbital bar. This configuration makes the anterior margin of this bar slightly inset from the outer orbital surface of the jugal. In addition, the postorbital bar does not have the sculptured pattern present in surrounding surfaces. The lateral surface of this process has a long ridge that spans the entire length of the infraorbital process at approximately midheight (Figure 10). The ridge, which we call the infraorbital ridge, arises anteriorly at the level of lacrimal and extends posteriorly to the round posterior (infratemporal) ramus. A longitudinal depression extends dorsal to this ridge from the anterior third of the orbital length and ends posterior to the postorbital bar. At both ends, this depression graduates to the jugal surface. A broad, fan-shaped, shallow depression occurs ventral to the ridge. Indeed, the ridge is a combination of a lateral hypertrophy of the jugal and the occurrence of shallow depressions dorsal and ventral to it. The surface of this depression is strongly sculpted and presents a reduced series of neurovascular foramina at its ventral portion. The ventral margin of the ventral depression is formed by a thin, lateral ridge at the ventral margin of the jugal. This thin ridge lies on the jugal-ectopterygoid suture and makes this suture visible in lateral view. This suture extends posteriorly towards the junction of the infraorbital lateral ridge with the infratemporal ramus of the jugal. The posterior portion of the depression, ventral to the infraorbital ridge, opens into a thin, deep notch. This notch separates the laterally facing jugal-ectopterygoid suture and ridge from the remainder of the jugal. The slender postorbital bar arises from the posterior third of the total length of the jugal body. The jugal is overlapped by the postorbital laterally at about mid-height of the postorbital bar. The infratemporal process is less than half as deep as, but transversely thicker than, the infraorbital process and forms the ventral margin of the infratemporal fenestra. Anteriorly, the infratemporal ramus of the jugal has a subquadrangular cross section. This shape is a result of the presence of two longitudinal depressions on its outer surface. Dorsally, the bone is marked by the posterior extension of the dorsal longitudinal depression of the jugal infraorbital ramus, and ventrally the jugal is marked by a longitudinal shallow groove starting just behind the ectopterygoid-jugal suture and ending at the midpoint of the infratemporal jugal ramus. Posteriorly, this cross section becomes cylindrical due to the absence of those depressions and slight increase of the diameter of the cross section.

An anterior suture for the nasal is preserved on the right premaxilla of LPRP/USP 0018. The suture indicates that the nasal would have entered the posterior midline of the external nares and likely formed at least a posterior division of the nares. The posteriormost portions of the nasals are preserved in LPRP/USP 0019. The bones are thin, anteroposteriorly elongated elements and participate only in the dorsal portion of the skull. They are fused to each other along most of their extension and have a wide dorsal surface. At the level of the anterior orbital margin, the nasals are dorsally concave, forming a low surface with regard to the remaining portion of the skull roof. The preserved portions indicate a tendency for the lateral margins of the concavity to smoothly converge anteriorly. Sutures between the nasal, prefrontal, and frontal are obscured by heavy sculpting, but there is certainly no nasal-frontal contact posteriorly, at least externally. The posterolateral corner of the nasal concavity forms the upper portion of the stepped prefrontals, which bounds the large anterior palpebral facet.

The rhomboidal prefrontals form the anteromedial margin and part of the anterior wall of the orbit. They have a large dorsal exposure, forming different surfaces on the medial and lateral halves that are separated by a step. The medial triangular surface is anteroposteriorly elongated and strongly ornamented with ridges and furrows. At the midlength of this portion, the major medial extension of the prefrontal contacts its counterpart and prevents contact between the frontal and nasal. The prefrontal contacts the frontal along a large, oblique suture. The lateral surface of the prefrontal is smaller and less ornamented. It has a broad articulation with the anterior palpebral bone. The right palpebral is missing, revealing the dorsal surface of the lateral portion of the prefrontal. This region is deeply excavated to form a broad articular facet that receives the anterior palpebral. The lateral margin of the prefrontal contacts the lacrimal along a nearly linear longitudinal suture. The prefrontal contributes to a nearly vertical orbital anterior wall. The prefrontal extends ventrally as a thin sheet of bone that forms the prefrontal pillar (Figure 9). The prefrontal pillar is transversely wide and spans from the midline to nearly the anterolateral margin of the orbit. The pillar is anteroposteriorly thin and posteriorly convex. Its anterior surface is not visible, but appears to have been concave and housed a pneumatic recess. The median portion of the prefrontal pillar extends to the pterygoid as a thin, flattened column arising from the ventromedial tip of the convex portion in the orbit. The column is ventromedially directed and expands distally in a semilunar shape oriented almost anteroposteriorly. The distal process barely contacts the pterygoid at its anterior tip, and no differentiated structure for its reception is observed on the palatine or pterygoid.

The lacrimal has two different surfaces on the skull. The dorsal portion is accessible on the right side contacting the prefrontal support for the palpebral via a linear, but jagged suture. This surface is almost unornamented and ventrally roofs the lateral surface of the lacrimal. The lateral surface of the lacrimal makes up much of the anterior margin and wall of the orbit. The ventral portion of the bone is firmly sutured to the dorsal region of the infraorbital process of jugal. This contact has a curved profile, which is continuous to the contact with the maxilla. The lacrimal, together with the curvature of the internal surface of prefrontal, also forms the lateral portion of orbital anterior wall. At the inflexion point between its dorsal and the lateral surfaces, the lacrimal is pierced by the foramen for the nasolacrimal duct.

The fused frontals have a complex outline. The anterior margins converge anteriorly, the lateral edge is concave about the orbital margin, and the posterior margin is convex, with the midregion bowing posteriorly. The anterior third is markedly ornamented and bears a smooth, midline depression (dorsal valley *sensu* [14]). The midline depression broadens posteriorly to form a dorsally concave surface over much of the remainder of the frontals. The lateral margins of the depression are slightly ornamented but are not raised to form an obvious ridge at the orbital rim. An unornamented sagittal crest, approximately 3 mm tall, occurs from the level of the posteriormost extension of prefrontal to near the frontal-parietal contact. The anterolateral portion of the frontal has a deep fossa for contact with the anterior palpebral. The frontal has a minor participation in the external supratemporal fenestra. The bone accompanies the thickened rim of the fenestra and forms a small anterior portion of supratemporal fossa. This part of the frontal is wedged between the postorbital and the parietal. Posteriorly, the frontal meets the parietal along an almost transverse suture, which extends between the medial thickened margins of the external supratemporal fenestrae. Posteroventrally, the frontal extensively contacts the dorsal portion of the laterosphenoids. Matrix obscures the ventral midline of the frontal and prevents access to the fine morphology of the cristae cranii and the passage for olfactory and optic traits. The posteroventrolateral corner of the frontal is underlapped by the postorbital but is entirely exposed laterally.

The fused parietals form the medial margin of the external and internal supratemporal fenestrae and fossae. This area is transversely constricted, and the medial margins of the external supratemporal fossae have distinct rims separated by a conspicuous sagittal groove. Anteriorly and posteriorly, the rims curve laterally and become continuous with the rims of the frontal, postorbital and supraoccipital. Within the supratemporal fossa, the parietal contacts the postorbital ventrally, preventing the frontal from bordering the internal supratemporal fenestra. The medial wall of the supratemporal fossa is formed by the parietal and is almost vertical and unornamented. The posteromedial part of this fossa is dorsoventrally deeper than other regions as a consequence of a gradual dorsal curvature of the parietal posteriorly. Posterolaterally, the parietal contacts the squamosal at the posterior portion of supratemporal fossa, at about the midpoint of the posterior margin of the internal supratemporal fenestra. A large oval temporo-orbital foramen is located on the suture between these two bones. This foramen faces dorsally and is confluent with a restricted groove in the parietal posterolateral portion. The posterior margin of that bone is firmly attached to the supraoccipital, forming an interdigitated suture along the parietal's entire transverse length.

The postorbital forms the anterolateral corner of the skull roof and possesses a descending process that forms the dorsal half of the postorbital bar. Dorsally, it also forms the posterolateral margin of the orbit as well as the anterolateral margin of the internal and external supratemporal fenestrae and fossae, separating the orbit from these other openings. The anterolateral corner of the postorbital bears a facet for the reception of the posterior palpebral. This facet is curved, and its lateral margin forms a short, sharp projection into the orbit. The auditory fossa is divided into two distinct portions. An anterior portion lies on the lateral surface of the postorbital and is separated from the posterior portion by a ridge at the quadratojugal-quadrate suture. This anterior portion extends to the posterior part of the postorbital bar, forming a posterolaterally oriented concavity at the back of the bar (Figure 14). A dorsolateral shelf, formed by the postorbital anteriorly and the squamosal posteriorly, overhangs the postorbital descending flange. This flange is a thin posteroventral extension of the postorbital body that overlies a small anterior portion of the quadratojugal. The slender postorbital bar is transversely oval in cross-section, and the postorbital bone reaches the mid-height of the bar, overlying the jugal ascending process laterally. Posterodorsally, the postorbital body is firmly attached to the squamosal at the level of the dorsal apex of the infratemporal fenestra.

The squamosal comprises the posterolateral corner of the skull roof. This element is slightly convex dorsally and has a distinctly sculptured dorsal surface. The ornamentations include a distinct pebbled surface at the border of external supratemporal fenestra. In lateral aspect, the posteroventral edge of the squamosal curves over the otic recess. This ventrally arched surface bears a shallow longitudinal sulcus, which was probably associated with attachment of muscles associated with an external earflap, as seen in extant and fossil crocodyliforms [9], [34], [48]. Anterodorsally, the squamosal is sutured to the postorbital nearly along the posterior third of the total length of the external supratemporal fenestra. The squamosal extends anteroventrally for a short distance, where it is overlapped by the descending flange of postorbital. Posteroventrally, the squamosal contacts the quadrate in front of the quadrate fenestrate area (Figure 14), but the posteroventral end of this contact is hidden by sediments and fractures. Posterodorsally, the squamosal deflects ventrally and extends posterolaterally from the skull roof, but the bone's lateralmost portion is not preserved. The posteroventral portion of the squamosal forms an inclined plate that extends posterolaterally, parallel to the dorsal edge of the paroccipital process. The anterolateral surface of this wall encloses the posterior margin of the external auditory meatus and the otic recess. The lateral margin of the dorsal portion of the squamosal extends over the auditory meatus. This arrangement creates a laterally opened auditory meatus, which is hidden in occipital view.

The quadratojugal forms nearly the entire posterior margin of the infratemporal fenestrae. The quadratojugal is unornamented, except for its anteroventral portion. The narrow anterodorsal ramus has a long sutural contact with the quadrate, which becomes distinctly ridged dorsal to its midlength. This ridged suture divides the otic recess in two different portions. The dorsal ramus of the quadratojugal is overlapped by the postorbital to exclude the quadrate and squamosal from the dorsal margin of the infratemporal fenestra. The infratemporal margin of the quadratojugal is smooth and lacks a spina quadratojugalis. A slight curvature is present at its dorsal midheight and may indicate the attachment of the musculus levator bulbi [9]. The posterior corner of the infratemporal fenestra is formed by the quadratojugal posterodorsally and jugal anteroventrally. The posteroventral portion of the quadratojugal extends a reduced process towards the mandibular condyle. In lateral view, this extension forms a small notch at the level of quadratojugal-quadrate contact (Figure 14). A lateral depression on the quadrate body extends onto the ventral portion of the quadratojugal-quadrate suture. The medial surface of the quadratojugal has long dorsal process, which prevents the contact between quadrate and postorbital, and reaches the ventral edge of the internal supratemporal fenestra.

The large quadrate contributes to the jaw joint and is sutured to the lateral wall of the braincase. The bone is anterodorsally inclined so that its distal articular surface is situated posterior and ventral to the occipital condyle but laying at the same level of the tooth row. The articular surface is transversally oriented. The surface is medially constricted and divided into two condyles. The medial condyle is ventrally developed and faces ventrolaterally. The lateral condyle is more dorsally positioned and posteroventrally oriented. This arrangement forms a sharp angle between the articular surfaces of each condyle. A dorsal extension of the intercondylar constriction is developed over the medial condyle. This extension is associated with a low crest and shallow groove that extends from the condyle to the contact between the quadrate and the paroccipital process near the cranio-quadrate passage. A well developed foramen aërum is situated on the posterior surface of the quadrate, near the dorsal margin of the condylar constriction (Figure 5), and opens into the shallow groove extending from the constriction.

The large otic aperture is laterally facing and has an elongated elliptical outline that is obliquely oriented in relation to the skull roof. The anterior and ventral margins of the otic foramen are entirely placed within the quadrate, and the dorsal and posterior margins are not visible. Four other apertures are present within a recessed fenestrate area of quadrate. These apertures border the ventral and anterior margins of the otic foramen and were probably external apertures of pneumatic quadrate diverticula. The posteriormost aperture is located ventral to the otic foramen, two apertures are anterodorsal to this ventral aperture, and the fourth aperture is positioned anterodorsally, in front of the otic foramen. The form and size of the apertures vary significantly; the posterior one has an elliptical shape and is the largest, i.e., about two thirds of the otic foramen size (20 mm in diameter). The other apertures are rounded and smaller, with the two ventral ones about 5 mm in diameter and the anterodorsal one about 9 mm in diameter. The orientation of the apertures is also variable. The ventral aperture faces posterodorsally, the anterodorsal aperture opens posteroventrally, and the two ventral apertures face more posteriorly. Ventromedially, the quadrate extends along the anteroventral edge of the paroccipital process to contact the basioccipital, basisphenoid, and pterygoid. The midlength portion of the contact between quadrate and basioccipital forms a low crest that is continuous to another crest extending from the medial margin of the medial quadrate condyle. This crest is similar to crest ‘D’ of [40]. Anteriorly, the quadrate contact against the braincase forms much of the ventral and posterior margins of the large trigeminal foramen. Anteroventrally, the quadrate is attached to the quadrate ramus of the pterygoid and to the posterolateral portion of the basisphenoid. Dorsomedially, the quadrate extends into the internal supratemporal chamber, contacting the ventral edges of the parietal and squamosal in the posterior wall of the internal supratemporal fenestra. The quadrate is firmly attached to the laterosphenoids anteriorly along a nearly vertical suture. Two low parallel crests occur lateral to the quadrate-pterygoid contact. The anterior crest is well defined and lies on the margin of the ventral and anterior portions of the quadrate (‘crest B’ of [40]). The other is less conspicuous and posteriorly adjacent. A large sigmoidal muscle attachment crest lies just posterior to the medial suture between quadrate and quadratojugal and probably corresponds to ‘crest A’ [39], [40] in extant crocodylians. This was related to the origin of the musculus adductor mandibulae posterior [9], [39], [40] or musculus adductor mandibulae externus superficialis [49].

Two palpebral bones are preserved in the left side of LPRP/USP 0019. They are thick elements with strongly ornamented dorsal surfaces. The anterior bone is hooked-shaped and articulates onto the prefrontal and lacrimal lateral projections, fitting on the step between those structures and the skull roof. Its posteromedial portion also fits in a small facet anterior on the orbital margin of the frontal. The anterior palpebral is dorsally convex, following the dorsal curvature of the skull at this level. Its posterolateral extension contacts the posterior palpebral about the level of the mid length of orbit. The posterior palpebral is small, well ornamented, and has a dorsally convex transverse profile. It has a subquadrate outline, articulates into a postorbital facet for this bone and contacts the anterior palpebral by means of a short spur of its anterolateral corner.

The palatine palatal shelves of LPRP/USP 0019 are tightly sutured into a stout element that forms nearly the entire posterior half of the secondary bony palate along most of its length (Figure 11). The palatine bar has a transversely reduced, ventrally flat, and extensively sculpted surface. Dorsally, the palatine becomes cylindrical with a nearly entirely unsculptured surface. The palatine-maxilla contact forms a transverse, interdigitaded suture. The suture traverses about one third of the palatal surface and is located just posterior to the anterior margin of the suborbital fenestrae. This arrangement prevents the palatine from reaching the lateral border of the suborbital fenestra near the tooth row. The midline suture is nearly straight along its entire length when viewed ventrally. The suture is ridged along its midlength and is flanked by a parasagittal groove in each side. The grooves become confluent posterior to the ridge. This confluence, combined with raised rims at the medial margins of the suborbital fenestra, forms a wide median depression on the posterior half of palatine palatal surface. A row of foramina dots the medial edge of the raised rim of the suborbital fenestra (Figure 11). The palatine extends a stout posterolateral process that is firmly attached to the anteromedial process of the ectopterygoid. This posterolateral processes bounds the anterior apex of the wide choanal aperture. The anteriormost portion of the palatal contact between the palatine and pterygoid is located anterodorsal to the anterior border of the choanal opening. The posterodorsal portion of the palatine is not accessible. Hhowever, it contacts a thin sheet of bone of the pterygoid (described below). This suture originates at the anterior tip of the dorsal surface of the parachoanal fenestra (see below) and extends perpendicularly to the sagittal plane for a short distance, then turning to an anterodorsal inclination in relation to the main palatal plane. Posterolaterally, the palatine sutures to the ectopterygoid at the anterior quarter of the lateral edge of the parachoanal fenestra. This dorsal portion of palatine is overlapped dorsally by the pterygoid and does not contact the prefrontal pillar. Together with the dorsal portion of the pterygoid, the palatine forms an inclined plane that roofs the posterior portion of narial passage.

The fused pterygoids have three distinct regions: a compact body attached to the anteroventral and lateral portions of the neurocranium, a pair of posteroventrally projecting flanges (pterygoid wings), and a broad, transversely concave and posteroventrally inclined sheet of bone bearing the choanal groove and parachoanal structures (choanal septum, fenestra and fossa, Figure 12). The anterior ramus of the pterygoid extends far anteriorly to the prefrontal pillar contact and is broadly exposed dorsal to the palatine when viewed through the orbit. Posterior to the contact with the prefrontal pillar, the pterygoids form a thin, sagittal septum that extends a short distance dorsally as an interorbital septum and continues posteriorly to suture to the parasphenoid. The pterygoid-parasphenoid dorsal margin is slightly broken, but appears to have been a single, continuous margin. There is no clear separation between the pterygoid and parasphenoid. A subtriangular fenestra occurs in the anterolateral portion of the pterygoid. We term this the parachoanal fenestra, because of its proximity to the choana. Dorsally, the anterior quarter of this fenestra is bounded by the palatine, the posterolateral half is bounded by the ectopterygoid, and the remainder by the pterygoid. Ventrally, this configuration remains the same, but the robust palatine bar, composed of the posterolateral ramus of the palatine and anteromedial ramus of the ectopterygoid, borders it anteriorly.

A large choanal depression is bounded anteriorly by the palatines, anterolaterally by the ectopterygoids, and the remainder entirely by the pterygoids. This depression houses a number of parachoanal structures and an elongated, divided choanal groove (Figure 12). The palatine-ectopterygoid margin of the choanae is ventrally located in relation to the remaining portion, so that the depression opens posteroventrally. The choanal septum, formed entirely by the pterygoids, is confluent with the posterior surface of the choana and is well recessed below the margin of the choanal depression. The ventral edge of the septum is subcolumnar in cross-section, with a distinctly flattened ventral surface. There is no trace of a midline ridge on the septum. The dorsal portion of the septum appears to be a thin sheet of bone. The posterior, medial, and lateral margins of the parachoanal fenestra extend onto the ventral surface of the pterygoids, as a broad fossa named the parachoanal fossa. This fossa is subdivided into medial and lateral subfossae by a smooth, low ridge. The left ridge only partly divides the medial and lateral portions of the parachoanal fossa, but the right ridge divides these portions nearly to the posterolateral margin of the parachoanal fenestra. The parachoanal fossae probably housed pneumatic chambers related to the choana and may have been used for vocalization. The medial parachoanal subfossa excavates a short distance into the pterygoid body. The lateral parachoanal subfossa excavates into the pterygoid body posteromedially and expands anterolaterally onto the ectopterygoid-pterygoid suture. The fossa expands anteriorly to excavate a chamber within the ectopterygoid body. At the lateral corner of this fossa, the ectopterygoid-pterygoid suture has a large, robust butt joint that extends to a nearly linear suture towards the tip of the pterygoid wing.

The pterygoid wings are overlapped by the ectopterygoids ventrally, which nearly reach the ventral tip of the pterygoid wing. The right pterygoid wing diverges at 80° and the left wing diverges at about 60° relative to the sagittal plane of the skull, and both wings diverge about 45° from the palatal plane, forming the ventralmost extension of the skull. The sheet of bone forming the parachoanal structures is thin and dorsally separated from the rest of pterygoid and ectopterygoid by a step, except along its posterolateral portion, where it is confluent with the pterygoid wing surface. This structure follows the ventral inclination of the pterygoid wings and culminates dorsally in the dorsal roof of the choanal aperture.

Behind the choanal opening, the pterygoid does not extend much ventrally, but forms two laterally displaced tuberosities confluent to the anterior portion of the basisphenoid. This configuration does not form a posterior vertical wall and clearly separates the pterygoid from the median Eustachian fossa anterior extension. Additionally, in the absence of a vertical posterior pterygoid wall, this area is not continuous to ‘crest B’ [39]. The contact between the pterygoid and quadrate extends dorsally along the braincase in a course that passes posterodorsally and then turns anterodorsally. The pterygoid extends up to the contact between the laterosphenoid and quadrate below the trigeminal foramen. From this point onward, the contact between the pterygoid and laterosphenoid extends anteriorly to the contact between the pterygoids. The base of this contact can be seen anteriorly, forming a sagittal crest in the interorbital space.

The posterodorsal process of the pterygoid extends until it contacts the quadrate ventromedial process at the level of the basioccipital lateral extension. The posterodorsal process is connected to the pterygoid wing via a curved blunt ridge. The medial portion of the pterygoid wing is very thin but becomes thicker laterodistally and has a pitted surface that presumably would have been covered by a cartilaginous cap in life. The thickness of the lateral and distal portions, combined with the ridged posteromedial dorsal edge, delimits a well marked depression that occupies mostly of the dorsal surface of the pterygoid wing (Figure 13).

The ectopterygoid is robust, caps the ventral edge of the pterygoid wing, and forms most of the posterior ventrolateral portion of the palate. On the pterygoid wings, the ectopterygoid descends to a point just short of the distal tip, and its ventral surface is ornamented with parallel longitudinal ridges. The body of the ectopterygoid has an elongate elliptical cross-section, with its long axis directed anteromedially. The ectopterygoid extends a small distance posteriorly onto the base of the pterygoid wing but does not reach the postorbital bar. The posterolateral contact with the jugal forms a right angle and is slightly raised from the ventral surface of the jugal. Foramina pierce the lateral surface of the ectopterygoid near the jugal contact. The right ectopterygoid bears five foramina of different sizes in this area, with the anterior one three times the size of any other, and the left ectopterygoid has only a single, large foramen. Anteriorly, the ectopterygoid has two small rami. The anterolateral ramus fits into the maxillary posterior process at the level of the midpoint of the suborbital fenestra. The stout anteromedial process overlaps the pterygoid ventrally and contacts the palatine posterolateral process to border the choanal opening anterolaterally. The dorsal surface of the ectopterygoid body is horizontally placed and contacts the jugal along most of its extension. Medial to the jugal contact, the ectopterygoid is deeply concave, nearly forming a ventrally facing plane until the origin of the pterygoid wing. The ectopterygoid-jugal suture extends essentially along the corner between the lateral and ventral surfaces of the skull and is distinctly ridged. This ectopterygoid configuration makes it border the lateral posterior half and the posterior tip of the suborbital fenestra. Dorsally, the ectopterygoid sutures to the palatine posterolateral process, perpendicular to the medial and lateral edges of the palatine bar. This suture extends medially to border the posterolateral half of the parachoanal fenestra. Posteriorly, the ectopterygoid contacts the pterygoid dorsally to the base of the pterygoid wing, forming an anteromedially directed suture that reaches the parachoanal fenestra.

The unpaired supraoccipital is visible on the dorsal surface of the skull as a thin slip articulated to the posterior margin of the parietal. This element tapers laterally until its inflexion to the occipital surface at the level of the medial edge of the temporo-orbital foramen. This dorsal exposure possesses a median depression confluent with the sagittal groove on the intertemporal bar. From its inflexion point, the supraoccipital is attached to the squamosal via a dorsally arched suture that contacts the otoccipital ventrally. In occipital view, the supraoccipital is wider than tall and forms a slightly posteriorly concave vertical wall. This surface bears a low median ridge restricted to the dorsal half of the bone, which is flanked on both sides by a depression that probably served for the insertion of nuchal ligaments. Additionally, the supraoccipital bears a series of radiating ridges which tend to converge ventrally towards the foramen magnum, forming a broad, triangular, ventral extension of the supraoccipital. The lateral articulation of this ventral extension is nearly linear. Ventrally, the supraoccipital is excluded from the dorsal margin of the foramen magnum by a broad median contact between the otoccipitals.

The laterosphenoid is a compact bone firmly attached to the anterodorsal portion of the braincase. It has a slender capitate process that diverges laterally from the basisphenoid body, reaching the postorbital. Its lateralmost portion forms a condyle that fits into a medial notch of the postorbital. The tiny tensor crest (probably related to musculus levator bulbi dorsalis, [43]) is distinguished in the median portion of the capitate process. The anteromedial extension of the capitate process extends onto the frontal. Posterior to the postorbital, the laterosphenoid probably contacted the parietal below the internal supratemporal fenestra. Posteriorly, the laterosphenoid is attached to the anterior portion of the quadrate just posterior to the foramen for passage of nervus trigeminus (V). The laterosphenoid forms the anterior and dorsal, as well as much of the posterior margins of the trigeminal foramen by means of a blunt ventral extension of the capitate process that is slight bifurcated at the level of this foramen. Anterolateral to this ventral extension, there is a rounded depression which probably enclosed the ophthalmic (V1) and oculomotor (fIII) foramina [43]. The well developed laterosphenoid ventral ridge probably overlaps the prootic laterally, but the suture is not visible.

The exoccipital and opisthotic are indistinguishably fused into a single otoccipital. The otoccipitals meet medially and form a bony shelf roofing the foramen magnum, as well as the lateral portion of this opening. Each element forms a broadly convex posterior surface on the occiput. That surface is dorsoventrally thin dorsal to the foramen magnum, but expands laterally, which combined with the dorsal extension of the depression on the medial condyle of the quadrate, forms a broad concave region distally and ventrally on the skull occipital plane. The otoccipital also contributes to the dorsolateral corner of the occipital condyle. The best preserved left side, near the foramen magnum, bears a single, small, posterolaterally facing hypoglossal foramen. Lateral to the hypoglossal foramen there is a larger, undivided, and posterolaterally facing vagal foramen, which would have provided route for nervi glossopharyngeus and vagus (IX–X). A small cranio-quadrate passage occurs ventral to the paroccipital process and medial to the triple contact between the squamosal, quadrate, and paroccipital process. This passage lies in the dorsal portion of a well marked medial depressed area of the occipital plane. The passage is partially covered by sediment, but extends medially into a cranio-quadrate canal running along the otoccipital-quadrate contact. The posterior surface of the distal end of the paroccipital process closes the ventral portion of the auditory meatus and bears distinct striations along its distal margin. These are possibly related to insertions of the musculus longissimus capitis superficialis and musculus iliocostalis capitis [44].

The basioccipital forms most of the peg-like occipital condyle, the external surface of which is not well preserved. Yet, it is clear that the basioccipital forms only the medial portion of the floor of the foramen magnum and cranial cavity. The basioccipital's posterior surface is slightly inclined and faces posteroventrally. The posteroventrally curved condylar neck is ornamented with tiny shallow pits and strongly deflected from the posteroventral surface of the basioccipital. The posteroventral surface of that bone slopes anteroventrally, forming an angle of approximately 30° relative to the skull roof plane. A short, median crest occurs from the base of the condylar neck to the opening of the median Eustachian tube (foramen intertympanicum), which is situated within a deep depression on the suture between the basioccipital and basisphenoid. The openings of the lateral Eustachian canals are lateral and slightly posterior to the median opening. The lateral openings are much larger than the medial foramen and have elongate, elliptical outlines (Figure 15). These openings lack a portion of their anterior walls so that the foramina open anterolaterally. The ventrolateral portions of the basioccipital are concealed by the basisphenoid and quadrates.

The basisphenoid and parasphenoid appear to be indistinguishably fused. The basisphenoid is largely exposed in ventral aspect and is inclined anteroventrally, following the slope of the basioccipital. The basisphenoid is bounded by the pterygoids anteriorly and basioccipital posteriorly, and the element's lateral projections contact the pterygoid ramus of the quadrate. The ventral surface of the basisphenoid has two short, but well defined, crests oriented parasagittally on the lateral margins of the median Eustachian tube (Figure 15). These crests delimit a deep groove between them, which is divided anteriorly by a transverse, less marked crest. Laterally, the basisphenoid forms a dorsal level which is separated from the ventral surface of quadrate and pterygoid by a well-developed step. As a consequence, the basisphenoid central region projects ventrally. The anterior contact with the pterygoid occurs posteriorly to the anterior rugose tuberosity that forms the ventralmost portion of the posterior wall of the choanae. The great ventral development of the basisphenoid and the occurrence of distinct ridges and tuberosities are possibly related to the insertions of the musculus longissimus capitis profundus and musculus rectus capitis anticus major, which are attached to the basal tubera in extant crocodyliforms [44].

### Mandible

Both partial mandibular rami of LPRP/USP 0018 are preserved and comprise much of the dentaries and the anterior part of the splenials (Figure 16). The number of dentary teeth is not determinable due to the complete overbite of the upper dentition. The mandibular symphysis is well developed and extends posteriorly until the level of the third maxillary tooth. The splenials form nearly the entire length of the strictly ventral surface of the symphysis. Anterior to that, the mandible is anterodorsally directed, at an angle of about 45°, and the symphysis is formed solely by the dentaries. In ventral view, the dentary-splenial suture tapers anteriorly, wedging the dentaries at the level of the divergence of the mandibular rami (Figure 16). Posteriorly, that suture extends subparallel to the medial edge of the mandible, but becomes gradually closer to that medial edge, and then turns onto the medial surface. This inflexion point occurs posterior to the symphysis, where the splenial covers the medial surface of dentary, probably bordering the tooth row medially. Behind the posterior end of the symphyseal facet, the splenial has a large slot-like foramen intramandibularis oralis for the mandibular ramus of nervus trigeminus (V3). The posterior margin of the symphysis is excavated by depressions, forming two distinct steps. The anterior step is at the level of the dentary-splenial suture, forming a narrow dorsally displaced surface. This depressed area is further dorsally displaced by a second, posterior, and more marked depression at splenial ventral surface. The area posterior to the anterior depression includes the ridged medial suture of the splenials, which more posteriorly forms a peg at the posterior surface of the symphysis. This depressed area is marked at its median portion by a pair of rounded distinct fossae that face posteroventrally. The outer surface is markedly sculptured at the anterior and ventral portions of the dentary. In addition, the splenial ventral surface is sculptured at the area between the ventral symphyseal depressions. The dentary is dorsoventrally deep along its entire preserved portion, especially at the level of the dentary caniniform tooth. At this point, the dentary also bulges laterally. The combination of the upturned dentary tip, the dorsoventrally deeper anterior portion, and the bulged lateral portion of the dentary, makes the anterior portion of mandible very robust. The second and third premaxillary teeth occlude onto slight notches on the mesial and lateral surfaces, respectively. The first alveolus is slightly procumbent, and the tooth occludes into a depression on the anterior portion of the premaxillary palatal shelf.

### Dentition

*Pissarrachampsa sera* has a reduced number of teeth, with only three in each premaxilla and four in each maxilla. The complete dentary tooth row is not preserved, but we estimate that it had more than 8 teeth. The premaxillary and anterior dentary teeth posses round cross-sections and are slightly posteriorly curved, lacking well-defined carinae. The maxillary and posterior dentary tooth crowns, on the other side, point backwards, and are strongly laterally compressed, with distinct, finely serrated mesial and distal cutting edges (ziphodont morphology, *sensu* [45]). The sizes of the teeth vary greatly; measurements are given in Table 3 and 4. The first and second premaxillary teeth are of similar size, whereas the third is hypertrophied, but all of them overhang the dentary mesially and laterally. The preserved external surface indicates that a deep occlusal pit between, as well as distal to, the first and second premaxillary alveoli received a dentary tooth (probably d1). When the mouth is closed, the maxillary teeth are placed in a lateral concavity of the dentary, so that m1 is slightly laterally displaced by the bulge of the anterior portion of that bone, which supports the caniniform tooth (d4). M2 is located in the ventralmost extension of the maxillary alveolar margin, so that its ventral tip is at the same level of the mandibular ventral edge.

**Table 3:** Maximum labiolingual and mesiodistal diameters, and apicobasal length (in cm) of premaxillary maxillary (m) tooth crowns of LPRP/USP 0019.

LPRP/USP 0019						
Cranial tooth position		Rigth			Left	
	Labiolingual	Mesiodistal	Apicobasal	Labiolingual	Mesiodistal	Apicobasal
m1	1.13	1.38	1.1	1	1.06	1.89
m2	2.2	2.15	4.54	1,5	2.0	2.22*
m3	1.35	1.22	1.67†	1,06	1.42	-
m4	1.03	1.0	-	0,68	0.98	0.76†

- Absent or not measurable,

† not fully erupted.

**Table 4:** Maximum labiolingual and mesiodistal diameters, and apicobasal length (in cm) of premaxillary (pm), maxillary (m) and dentary (d) tooth crowns of LPRP/USP 0018.

LPRP/USP 0018			
Cranial tooth position		Right	
	Labiolingual	Mesiodistal	Apicobasal
pm1	0.55*	0.8	0.99*
pm2	0.63	0.8	1.42
pm3	1.24*	1.22*	0.89*
m1	0.55*	0.74*	1.59*
m2	0.97*	1.46	3.88
m3	-	1.03*	1.32*
m4	-	0.61*	1.16*
d1	0.66	-	1.72
d4	0.9*	1.7	2.38*

*Incomplete or not fully measurable,

- absent or not measurable.

The serrated mesial and distal cutting edges of the teeth are better preserved in LPRP/0019. The serrations are placed along most of the crown mesial and distal margins (except at the tip, where they are probably worn off) and are formed by true denticles juxtaposed one another. In this sense, *Pissarrachampsa* has true ziphodont teeth *sensu* [45]. The morphology of the denticles, as well their size, does not vary significantly along the carinae. Each denticle is represented by a trapezoidal element with a sharp outer margin. As a consequence of this pattern, the density of denticles is almost the same along most of the carinae (3–4 denticles/mm).

## Results

### General comparisons among Baurusuchidae

*Pissarrachampsa sera* is obviously related to Baurusuchidae. The group has long been diagnosed in precladistic and cladistic studies by the presence of a laterally compressed rostrum, absence of antorbital fenestrae, notch in rostrum for the reception of dentary caniniform tooth, approximation of prefrontals, well developed palatine bar, ectopterygoids forming the choanal border, quadratojugal extending dorsally as broad sheet contacting most of postorbital portion of postorbital bar, vertical quadrate, reduced number of teeth, hypertrophied maxillary tooth and ziphodont dentition [1], [18], [19], [21], [45], [50]–[52]. All these traits are present in *Pissarrachampsa*, but within clade relationships have never been discussed. Although baurusuchids are easily distinguished from the other Mesozoic crocodyliforms, the clade posseses a relatively high degree of ingroup morphological variation that is often overlooked. We present a detailed diagnosis for each putative South American baurusuchid in Text S1 in order to determine a unique suite of characters for each species, justifying a broader phylogenetic analysis of baurusuchids. These diagnoses validate the discrete taxonomic assignments of these taxa and may aid in future revisions of other possible baurusuchids. Before discussing specific characters, we provide additional comparisons within the group. These general comparisons are difficult to quantify and/or qualify as discrete characters and have not yet been used in phylogenetic analyses.

One of the most notable variations within the group is size. *Cynodontosuchus* is the smallest baurusuchid (but see following sections) with a preserved rostrum of less than 100 mm. In contrast, *Stratiotosuchus* has a skull length of approximately 470 mm. The relative proportions between different skull regions also vary. A most obvious example is the variation of rostral length. In *Pissarrachampsa*, the rostrum, measured from the anterior edge of the orbit to the tip of the premaxilla is estimated to be only 42% of the total skull length and falls into the “short rostrum category” of [38]. The rostra of *Stratiotosuchus* and *Baurusuchus salgadoensis* are nearly 65% of the skull length and classified as “normal”, close to “long” rostra. The robustness of the skull bones also varies, even though this trait is hard to quantify. *Pissarrachampsa*, *Wargosuchus*, *Baurusuchus pachecoi*, and *Cynodontosuchus* have more lightly built skulls compared to *Stratiotosuchus* and *Baurusuchus salgadoensis*, which have more massive skulls.

Orientation of the external nares, orbits, choanae, and distal quadrates also vary slightly within the group. This variation is not marked enough to warrant discrete character state coding, but may be useful in future analyses. The external nares of most baurusuchids open directly anteriorly. Those of *Stratiotosuchus* open in a more anterodorsal direction, with the dorsal roofing bones of the nares slightly retracted posteriorly.

The otic region is highly variable among baurusuchids. *Stratiotosuchus*, *Baurusuchus salgadoensis,* and *B. albertoi* have an enlarged otic recess and the descending flange of the squamosal does not cover much the otic aperture. In contrast, this flange partially hides the otic recess and the otic aperture in *Pissarrachampsa* and *Baurusuchus pachecoi*. The lateral depression on the quadrate varies from the anteroposteriorly broad depression in *Pissarrachampsa*, which expands onto the quadratojugal-jugal suture, to the dorsoventrally expanded but anteroposteriorly restricted depression in *Stratiotosuchus*, to the subcircular depression in other baurusuchids. The anterior support for palpebrals varies from the laterally stepped articulation with the prefrontal in *Pissarrachampsa*, *Wargosuchus* and *Stratiotosuchus* to an abbreviated step in *Baurusuchus pachecoi* and *Baurusuchus salgadoensis*.

### Phylogenetic Relationships

The phylogenetic relationship of *Pissarrachampsa sera* within Baurusuchidae was examined with a maximum parsimony analysis of discrete morphological characters. The analysis was based on a new dataset constructed to address the morphological variation observed in Baurusuchidae. The taxon sampling scheme was conceived to incorporate a broad diversity of Baurusuchidae, with the ingroup composed of seven putative South American baurusuchids scored at the specific level (*Pissarrachampsa sera*, *Baurusuchus albertoi*, *Baurusuchus pachecoi*, *Baurusuchus salgadoensis*, *Cynodontosuchus rothi*, *Stratiotosuchus maxhechti* and *Wargosuchus australis*). *Notosuchus terrestris*, *Mariliasuchus amarali*, and *Armadillosuchus arrudai* were used as outgroups. The data sources for each taxon are given in Text S2. Some taxa that have been suggested to be baurusuchids or closely related to baurusuchids were excluded from the analysis. These include *Bergisuchus dietrichbergi* [26], *Iberosuchus macrodon* [24], *Eremosuchus elkoholicus* [25], *Pabwehshi pakistanensis* [17] and *Pehuenchesuchus enderi* [19]. These taxa were excluded a priori from the parsimony analysis because the baurusuchid affinity of these forms were repeatedly challenged within different phylogenetic frameworks of Mesoeucrocodylia [6], [8], [9], [12], [19], [28]–[31]. Moreover, their fragmentary nature prevents both the identification of the apomorphic traits of the South American baurusuchids and the proposition of a unique suite of characters that can diagnose them as discrete baurusuchid taxa. Firsthand inspection of the holotype of *Bergisuchus dietrichbergi* (Hessisches Landesmuseum -Me 7003) and *Iberosuchus macrodon* (Museu Geológico- 5679), as well as descriptive accounts of these taxa [24], [26], [53], identified antorbital fenestrae and a broad contact between the nasal and frontal in *Bergisuchus* and five premaxillary teeth in *Iberosuchus*. Those characters are not present in any of the more complete undoubted South American baurusuchids. In the same way, the firsthand analysis of the holotype of *Pehuenchesuchus enderi* (Museo Municipal Argentino Urquiza -PV-CRS-440), along with its descriptive accounts [19], and the descriptive accounts of *Eremosuchus elkoholicus* (25) revealed the presence of sixteen alveoli in the dentary of *Pehuenchesuchus* and twelve in *Eremosuchus*, which is not congruent with the ten alveoli found in the dentary of the more complete undoubtful baurusuchids. *Pabwehshi pakistanensis* was excluded because it has a sagittal torus on its maxillary palatal shelves [9], absent in the putative baurusuchids.

Although many early and current analyses have recovered a close relationship between Baurusuchidae and Sebecidae, this is based on only six discrete characters: i.e., a deep rostrum, a well developed notch in premaxilla-maxilla suture, lateral surface of the dentary with longitudinal groove, a hypertrophied dentary tooth, ziphodont dentition, and a sigmoidal tooth row in dorsal view [19], [34]. Recent analyses have challenged this hypothesis, also based on broad taxonomic and morphologic samplings [9]. Moreover, characters that distinguish the outgroup of the present analysis and baurusuchids outnumber the putatively shared traits between baurusuchids and sebecids, and few of the outgroup characters are present in sebecids. A detailed discussion of these aspects is presented below. A more detailed analysis of the sebecid-baurusuchid relationship is beyond the scope of the present work.

The outgroup was constructed based on the notosuchian affinity of Baurusuchidae [6], [54] accepted in most recent phylogenetic analyses [7]–[12], [15], [19], [30], [54]–[57]. Three taxa were used based on the completeness of material and their possible proximity to Baurusuchidae, estimated by the coding of the new characters presented here. *Notosuchus terrestris* [23] is used as the primary outgroup taxon and *Mariliasuchus amarali* [58] and *Armadillosuchus arrudai* [59] were added to promote a better resolution of character transformations through basal nodes of the ingroup.

**Figure 17:**
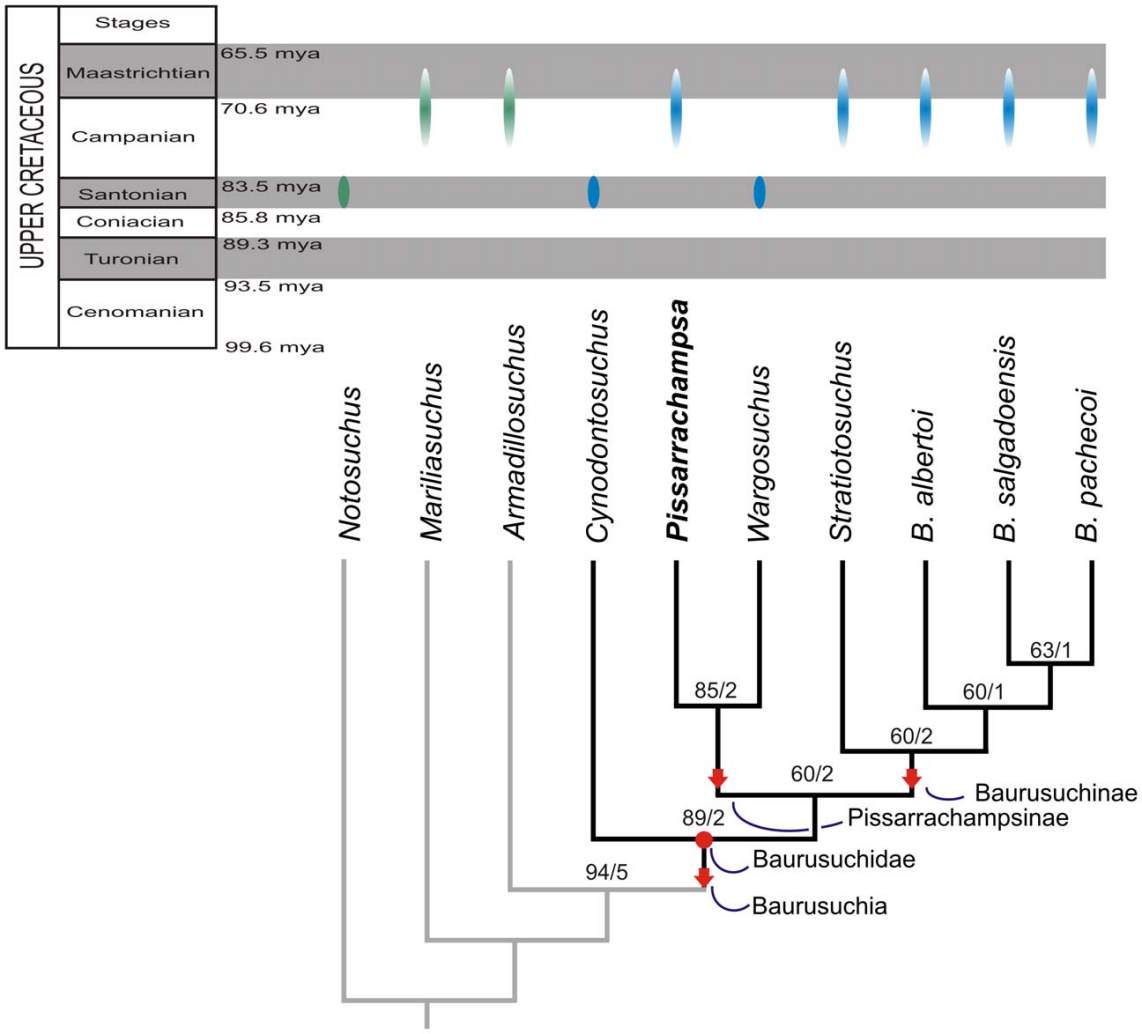
Phylogenetic relationships of Baurusuchidae depicting the position of *Pissarrachampsa sera*. The single most parsimonious tree (total length  =  80 steps; consistency index  =  0.83; retention index  =  0.84) recovered after maximum parsimony analysis using TNT. The bootstrap (50% cut) and decay values are shown above the clades. Ages from [21], [105] for Argentinean taxa and [101], [102] for Brazilian taxa. Arrows assigned to stem-based taxa, and circle for node based.

Characters were selected focusing on the internal relationship of Baurusuchidae. Thirty-five new characters were added to a set of characters that previous mesoeucrocodylian phylogenetic analyses have recovered as synapomorphies of different clades (see Text S3). Autapomorphies for the terminal among are included in the list of character and in the matrix, but excluded at the analytical stage. The autapomorphies are included to strengthen the unique condition of the taxa, but also to be available for testing by further, broader Mesoeucrocodylia phylogenetic analyses. The matrix composed of 10 taxa and 66 characters (Text S4) was analyzed using the software TNT 1.1 [60] under the implicit enumeration algorithm. Different indices were calculated to verify the internal consistency of the data. In addition to consistency and retention indices for the tree topology, we performed a standard bootstrap analysis (1000 heuristic replicates) and calculated the decay index [61] for nodal support (see Figure 17).

## Discussion

### Tree Topology

The maximum parsimony analysis yielded a single tree (Figure 17) with 80 steps; see discussion below and Text S5 for the optimized synapomorphy list. The topology places *Mariliasuchus amarali* as the sister group of *Armadillosuchus arrudai* plus a monophyletic Baurusuchidae. *Cynodontosuchus rothi* is the most basal taxon within Baurusuchidae, sister to a dichotomy leading to a clade composed of *Stratiotosuchus* and all *Baurusuchus* species and a clade composed of *Pissarrachampsa* and *Wargosuchus*. Within *Baurusuchus*, *B. albertoi* is the sister taxon to a clade composed of *B. pachecoi* and *B. salgadoensis*.

## Phylogenetic definitions

### Baurusuchidae Price 1945

#### Diagnosis

Small to medium-sized mesoeucrocodylians with premaxillae-maxillae suture internalized in a notch for the reception of lower caniniform; alveolar margin of maxilla in lateral view arched anterior to enlarged caniniform tooth; anterior extension of palatines not reaching the level of the anterior margin of suborbital fenestrae; presence of the posteroventral depressions in the mandibular symphysis; orientation of terminus of mandibular symphysis anterodorsal, at approximately 45 degrees to jaw line; teeth with serrated mesial and distal carinae.

#### Etymology

Baurusuchidae was established as a family name on the basis of *Baurusuchus pachecoi* Price 1945 [1].

#### Phylogenetic definition

The least inclusive clade containing *Cynodontosuchus rothi* Woodward 1896 [23], *Pissarrachampsa sera* **n. gen. n. sp.**, *Stratiotosuchus maxhechti* Campos, Suarez, Riff & Kellner 2001 [62] and *Baurusuchus pachecoi* Price 1945 [1], as long as it does not include *Notosuchus terrestris* Woodward 1896 [23], *Mariliasuchus amarali* Carvalho & Bertini 1999 [58], *Armadillosuchus arrudai* Marinho & Carvalho 2009 [59], *Araripesuchus gomesi* Price 1959 [63], *Sebecus icaeorhinus* Simpson 1937 [64], *Bretesuchus bonapartei* Gasparini, Fernandez & Powell 1993 [65], *Peirosaurus tormini* Price 1955 [66], or *Crocodylus niloticus* Laurent 1768 [67].

#### Discussion

The primary goal of this work is to examine baurusuchid ingroup relationships and determine a clear defifnition for Baurusuchidae (see Figure 17). The only available Baurusuchidae node-based definition [18] uses *Baurusuchus* and *Stratiotosuchus* as internal specifiers, and no external specifier was postulated.

*Cynodontosuchus* is historically important for Baurusuchidae because it was the first described member of the group [21], [23], and associated, or even claimed to be congeneric, with *Baurusuchus* in early and current literature [51], [52], [63], [68]–[70]. This compelled us to include *Cynodontosuchus* as an internal specifier in a phylogenetic definition of Baurusuchidae. Yet, if *Cynodontosuchus* turns out to be a juvenile specimen, or if its phylogenetic position within Baurusuchidae changes in future analyses, the defined clade remains stable (see below). We also included several other taxa as internal specifiers to better sample the morphological variation of the group. External specifiers were selected to avoid misapplication of the name in unlikely event that future alternative topologies recover those taxa as a paraphyletic assemblage.

### Baurusuchia Walker 1968

#### Diagnosis

Same as Baurusuchidae.

#### Etymology

The name was proposed [71], [72] as a modififcation of the familiar name created earlier [1] to the infraorder level to reflect the opinion of the author regarding the highly modified morphology of the holotype of *Baurusuchus pachecoi* Price 1945. We choose to respect this earlier name and revise its definition to reflect its original intention to demarcate the highly divergent morphology of the clade.

#### Phylogenetic definition

*Baurusuchus pachecoi* Price 1945 [1] and all Crocodyliformes that share a more recent common ancestor with *Baurusuchus pachecoi* Price 1945 [1] than with *Notosuchus terrestris* Woodward 1896 [23], *Mariliasuchus amarali* Carvalho & Bertini 1999 [58], *Armadillosuchus arrudai* Marinho & Carvalho 2009 [59], *Araripesuchus gomesi* Price 1959 [63], *Sebecus icaeorhinus* Simpson 1937 [64], *Bretesuchus bonapartei* Gasparini, Fernandez & Powell 1993 [65], *Peirosaurus tormini* Price 1955 [66], and *Crocodylus niloticus* Laurent 1768 [67].

#### Discussion

We propose Baurusuchia as a stem-based name to encompass Baurusuchidae, based on the large morphological gap between this clade and the outgroup (see below). In the present analysis, Baurusuchia is topologically identical to Baurusuchidae. Although these two taxa share the same diagnosis, our goal for this taxonomic decision is to minimize future, unnecessary taxonomic changes by providing a stable taxonomic framework for baurusuchian systematics.

### Pissarrachampsinae subfam. nov

#### Diagnosis

Medium sized baurusuchids with posterior portion of dorsal surface of the nasal bearing a rugose broad depression; approximation of prefrontals along their medial edges anteriorly; presence of a midline longitudinal depression on anterior portion of frontal.

#### Etymology

Named on the basis of *Pissarrachampsa sera* **n. gen. n. sp.**, the most complete member of the clade.

#### Phylogenetic definition

*Pissarrachampsa sera* **n. gen. n. sp** and all Crocodyliformes that share a more recent common ancestor with *Pissarrachampsa sera* **n. gen. n. sp** than with *Stratiotosuchus maxhechti* Campos, Suarez, Riff & Kellner 2001 [62], *Baurusuchus pachecoi* Price 1945 [1], *Notosuchus terrestris* Woodward 1896 [23], *Mariliasuchus amarali* Carvalho & Bertini 1999 [58], *Armadillosuchus arrudai* Marinho & Carvalho 2009 [59], *Araripesuchus gomesi* Price 1959 [63], *Sebecus icaeorhinus* Simpson 1937 [64], *Bretesuchus bonapartei* Gasparini, Fernandez & Powell 1993 [65], *Peirosaurus tormini* Price 1955 [66], and *Crocodylus niloticus* Laurent 1768 [67].

#### Discussion

The basal dichotomy of Baurusuchidae is well supported. Pissarrachampsinae is defined here as a stem-based clade to encompass its morphological unity as recovered in the present analysis.

### Baurusuchinae subfam. nov

#### Diagnosis

Medium sized baurusuchids with the medial contact for the prefrontals occurring along most of their dorsal medial edge; frontals broad, about twice the width of nasals; quadratojugal dorsal extension ending ventrally, or at the same level, of the dorsal tip of laterotemporal fenestra; quadrate fenestrae internalized in otic notch; muscle scar in the medial surface of quadrate (ridge ‘A’ [40]) almost straight to curved; ventral surface of choanal septum ridged.

#### Etymology

Named on the basis of *Baurusuchus pachecoi* Price 1945 [1], the first described member of the clade.

#### Phylogenetic definition

*Baurusuchus pachecoi* Price 1945 [1] and all Crocodyliformes that share a more recent common ancestor with *Baurusuchus pachecoi* Price 1945 [1] than with *Pissarrachampsa sera* **n. gen. n. sp.**, *Notosuchus terrestris* Woodward 1896 [23], *Mariliasuchus amarali* Carvalho & Bertini 1999 [58], *Armadillosuchus arrudai* Marinho & Carvalho 2009 [59], *Araripesuchus gomesi* Price 1959 [63], *Sebecus icaeorhinus* Simpson 1937 [64], *Bretesuchus bonapartei* Gasparini, Fernandez & Powell 1993 [65], *Peirosaurus tormini* Price 1955 [66], and *Crocodylus niloticus* Laurent 1768 [67].

#### Discussion

Baurusuchinae expands on the “practical use” of Baurusuchidae in previous phylogenetic analyses, which were limited to *Baurusuchus* and *Stratiotosuchus* (e. g: [29], [30], [33], [34], [73]–[75]) but see [31]. Here, we recognized this internal branch of Baurusuchidae as a well established, stem-based clade.

### Character Optimization and Discussion

The proposed phylogenetic hypothesis is used here as a template to describe the evolutionary history of the unique morphology of Baurusuchidae, addressing the synapomorphies of the group and its internal clades. *Cynodontosuchus rothi* and *Wargosuchus australis* bear some problems for a complete discussion of character evolution in the clade, because of their fragmentary nature and large number of missing entries in the data matrix. We evaluate ambiguous character transformations for these poorly scored taxa using accelerated (ACCTRAN) and delayed (DELTRAN) transformation [76], [77]. We agree that each case must be evaluated independently, and there is no overall best optimization method [78].

In the present dataset, alternative reconstructions of characters optimizations are more often caused by missing entries (characters 3, 5, 9, 11–14, 18, 19, 22, 23, 24, 27, 30, 31, 35, 37–41, 43, 45, 46, 48, 49, 51, 53, 54, 56, 58, 60, 61, 62, 64, and 65), and only three cases are caused by a combination of missing entries and conflicting scoring for different taxa (characters 16, 32, and 33). The use of ACCTRAN is these cases would imply the presence of characters that are in fact not preserved in various taxa. Therefore, for the following discussion we adopted DELTRAN as the optimization procedure. This is a more conservative approach for the case at hand, and assigns the synapomorphies to the clades that in fact show the derived states.

Baurusuchidae is diagnosed by seven synapomorphies (characters 1.1, 22.2, 40.1, 55.2, 56.1, 62.1, and 64.1; character states are given after each character number, separated by a period), including those recovered unambiguously, as well by the optimization process. The presence of the premaxillae-maxillae suture internalized in a notch for the reception of lower caniniform (character 1.1), the alveolar margin of maxilla in lateral view arched anterior to enlarged caniniform tooth (character 22.2), and the teeth with serrated mesial and distal carinae (character 55.2) are character-states with multiple origins within Crocodyliformes. The same conditions are found in “protosuchian”, Peirosauridae, Mahajangasuchidae, Eusuchia, and other taxa. The premaxilla-maxillary notch and the serrated carinae are important characters in the context of the monophyly/paraphyly of Sebecosuchia (see [9] for more information). The same conditions are found in *Sebecus*, *Bretesuchus,* and *Iberosuchus,* and are considered as ambiguous synapomorphies of Sebecosuchia, when this clade is recovered in the analyses (e.g.,[12], [13], [34], [79]). Yet, these characters represent ambiguous apomorphies for Baurusuchidae when Sebecosuchia is not recovered [30], showing that at some point the phylogenetic signal of these characters categorize Baurusuchidae. The palatine's anterior extension not reaching the level of the anterior margin of the suborbital fenestrae (character 40.1) and the presence of the posteroventral depressions in the mandibular symphysis are important new characters that represent delayed synapomorphies. The conditions found within Baurusuchidae are unique among Mesoeucrocodylia and are examples of the modified morphology of the group. The premaxillary dentition composed of three teeth (character 56.1) is a new apomorphy proposed for the group, which highlights its previously underestimated internal variation. A reduced dental formula is a general baurusuchid trait [1], [18], [62], but when the internal branch pattern is taken into account, it is clear that a posterior increase in the premaxillary tooth number characterizes *Baurusuchus*. The anterodorsal orientation of the mandibular symphysis terminal portion at an angle of approximately 45 degrees to the jaw line (character 62.1) is confirmed in the present analysis as a baurusuchid synapomorphy, as in previous works [30].

A recovered clade grouping the baurusuchids above *Cynodontosuchus* (Figure 17) shows three new unambiguous synapomorphies: ventral face of palatine bar restricted and dorsal portion cylindrical (character 41.1), rugose medial palatal contact (character 42.1), and a row of foramina flanking the medial contact of palatines (character 44.1). These unique morphological traits are synapomorphies grouping the higher Baurusuchidae apart from *Cynodontosuchus* and evidence for an important morphological gap between *Cynodontosuchus* and the other taxa. In addition, 18 extra delayed synapomorphies characterize this clade (characters 5.1, 9.1, 12.2, 14.1, 18.1, 19.1, 23.1, 24.1, 27.2, 31.1, 33.1, 37.1, 38.1, 45.1, 51.1, 54.1, 60.1 and 61.1). These characters are not scored for *Cynodontosuchus,* but are evidence for the huge morphological changes basal to, or within basal Baurusuchidae. These are a combination of characters previously recognized in the literature as synapomorphies at different levels of mesoeucrocodylian phylogeny, plus a great number of characters proposed here. Among them, the median approximation of prefrontal and the lateral depression of quadrate are tested here under maximum parsimony, turning to be phylogenetically relevant as previously proposed [21], [80]. In addition, a character previously discussed in the literature concerning baurusuchids is the presence of a triangular depression on the lateral surface of the jugal ([81]: character 133; [6]: character 145), deemed as a synapomorphy of sphagesaurids and Sebecosuchia (e.g.: [6], [13], [34]). Based on a close examination of baurusuchids and outgroup taxa, this character was split in two (characters, 27 and 45). The depression is interpreted here as a combination of two features: the displacement of the lateral surface of the infratemporal portion of the jugal, extending anteriorlly and reaching the orbital anterior edge, and the ridged ectopterygoid-jugal suture. The first state is a further modification of a feature also recognized in some outgroup taxa (*Mariliasuchus* and *Armadillosuchus*), as well other forms not included in the present analysis (e.g., *Sphagesaurus*, *Yacarerani*, *Adamantinasuchus*). The second character is unique to the clade. The combination of these two features, plus the infraorbital portion of the jugal being twice as large as the infratemporal portion, exacerbates the condition for Baurusuchinae and leaded to the oversimplification of the character in previous analyses.

*Pissarrachampsa sera* is positioned as sister clade to *Wargosuchus australis*, forming Pissarrachampsinae. This association is based on three synapomorphies: posterior portion of dorsal surface of the nasal bearing a rugose broad depression (character 4.1), prefrontal medial margins anteriorly convergent (character 5.1), and presence of a midline longitudinal depression on anterior portion of frontal (character 10.1). Among these characters, only the presence of the broad depressed area on the posterior portion of the nasal was not considered as an autapomorphy of *Wargosuchus* in its original description [21]. On the other side, the taxa differ based on a groove between the prefrontal and anterior palpebral dorsal surfaces (character 6.1) and a small contact between nasal and frontal, both present only in *Wargosuchus* [21], and the autapomorphic position of the lacrimal duct in *Pissarrachampsa* (character 20), contrasted to the plesiomorphic condition in *Wargosuchus*. Considering the fragmentary nature of *Wargosuchus*, the number of synapomorphies and differences are enough to respectively accept them forming a monophyletic group, but representing different genera.

Baurusuchinae shares medial contact of the prefrontals most of their dorsal medial edge (character 5.2); frontals broader, about twice the width of nasals (character 8.1); quadratojugal dorsal extension ending ventrally, or at the same level, of the dorsal tip of laterotemporal fenestra (character 32.0); quadrate fenestrae internalized in otic notch (character 34.1); muscle scar in the medial surface of quadrate (ridge ‘A’ [40]) almost straight to curved (character 36.0); and ridging of the ventral surface of the choanal septum (character 47.1). Additionally, two delayed character states diagnose this clade: the frontal longitudinal crest restricted to the posterior portion (character 11 state 1); and the quadrate depression main axis is dorsoventrally oriented (character 33 state 1). The presence of multiple quadrate fenestrae (or preotic siphonal foramina, *sensu* [15], [30], [82]) is known in protosuchians [13], [30], [34] and notosuchians, and is especially evident and studied in *Notosuchus* and *Mariliasuchus* (see [83]). In the present paper we recognized a fenestrate quadrate also in *Armadillosuchus* and Baurusuchidae. However, within Baurusuchidae, only *Pissarrachampsa sera* exposes these fenestrae in lateral view. Baurusuchines posses a modified condition, in which the four quadrate fenestrae are enclosed in the otic notch and only the anteriormost is partially visible in lateral view. The function of the quadrate fenestrae have not been discussed previously and is beyond the scope of this work, but a relationship to auditory function is possible.

*Baurusuchus* is diagnosed by an antorbital region of the jugal more expanded than the infraorbital region (character 25) and two delayed synapomorphies: major surface of pterygoid wing latero-ventrally oriented (character 48) and *musculus pterygoideous* posterior insertion in a marked depression on the surangular-angular lateral surface (character 61). *Baurusuchus pachecoi* shares with *B. salgadoensis* the ectopterygoid-jugal suture ridge continuous with the ventral ridge of the infratemporal portion of jugal (character 45) and three delayed synapomorphies: cylindrical dorsal portion of palatine constricted posteriorly (character 41 state 2), premaxillae bearing four teeth (character 56), and last premaxillary tooth not hypertrophied (character 58). The new interpretation of the jugal antorbital triangular depression (see discussion above) leads to the recognition of a ridged ectopterygoid-jugal suture. *Pissarrachampsa*, *Stratiotosuchus,* and *Baurusuchus albertoi* show this ridge ending in a well marked notch (see Figure 10), which separates this structure from the displaced lateral surface of the infratemporal portion of the jugal. In contrast, *B. pachecoi* and *B. salgadoensis* have a smooth transition between these ridges, and no notch is observed.

### Predictions for future stability of the nomenclatural acts proposed

Two predictions are considered here with possible consequences to the nomenclatural acts proposed. *Cynodontosuchus* has a unique suite of characters that seems best explained by advocating a distinct taxonomic assignment for it. However, alternative scenarios may also explain those differences and lead to another interpretation of its status, as the suggestion of *Cynodontosuchus* representing a juvenile *Baurusuchus* [51], [52]. In addition to its relatively small size, the presence of smoother bone sculpturing may be a result of fewer resorption-reconstruction cycles, which is also characteristic of early ontogenetic stage in many tetrapod groups [84]–[87]. Assuming this scenario, the absence in *Cynodontosuchus* of the palatal median rogosity and its flanking foramina rows, which is present in higher baurusuchids, can also be interpreted as a juvenile condition given that singular structures develop latter in ontogeny [84], [88]–[92]. The absence of hypertrophied teeth in the premaxilla can also be consistent with this alternative interpretation, because juveniles of extant Crocodylia tend to have teeth with less size differentiation ([84]; HCEL pers. observ.). In addition, the closely associated taxon *Notosuchus terrestris* also presents a progressive development of caniniform teeth during ontogeny [93]. The exact provenance of *Cynodontosuchus* is not known [52], [69], [94], but based on its association with *Notosuchus* [23], it is reasonable to assume an origin within the Bajo de La Carpa Formation [21], [29], [52], [69], [94], which also yielded *Wargosuchus*. The presence of both *Wargosuchus* and *Cynodontosuchus* in the same stratigraphic unit may suggest that they represent different ontogenetic stages of the same taxon, instead of two sympatric baurusuchids. If *Cynodontosuchus* represents a juvenile *Wargosuchus*, the Baurusuchidae node would move to the Pissarrachampsinae-Baurusuchinae dichotomy. The stem-based Baurusuchia was proposed to accommodate future new taxa that may lie outside Baurusuchidae but are clearly more closely related to the baurusuchid clade than the other notosuchians. New taxa are predicted to be found along this stem, because of the large morphological gap between known baurusuchids and their notosuchian sister groups. The stem-based definition will easily accommodates any future new taxa, maintaining the morphological unity of Baurusuchidae, and not pose further taxonomic rearrangements.

### Biostratigraphical Implications

*Pissarrachampsa sera* comes from the Triângulo Mineiro, in Minas Gerais, where the Vale do Rio do Peixe Formation has some of its northernmost surface exposures. This region has received less paleontological attention than the Marilia Formation near Peirópolis, also in Triângulo Mineiro, where a large number of tetrapod taxa have been recovered [66], [69], [95]–[99]. The Vale do Rio do Peixe Formation corresponds mainly to the Adamantina Formation in more conventional stratigraphic schema [100], which has been dated at Turonian-Santonian and Campanian-Maastrichtian from strata in São Paulo state [101], [102]. The latter date is more likely, given the correlation based on vertebrates to nearby Campanian-Maastrichtian stratigraphic units [102]–[104]. In the more southern São Paulo state, the Vale do Rio do Peixe Formation has yielded four baurusuchid species (Figure 2), whereas *Pissarrachampsa sera* is the only baurusuchid known from Minas Gerais. However, the stratigraphic correlation between these northern and southern exposures of the Vale do Rio do Peixe Formation has not been fully determined.

The phylogenetic results presented here have interesting biostratigraphic implications. Baurusuchines are restricted to the Vale do Rio do Peixe Formation of São Paulo state, suggesting that the four species were either living in sympatry, are in fact stratigraphically isolated, or a combination of both. *Wargosuchus* comes from the Bajo de la Carpa Formation, which has been assigned a Santonian age [21], [94], [105]. Pissarrachampsinae then either spans at least 20 million years, or corroborates the older age for the Vale do Rio do Peixe Formation [101], at least in Minas Gerais. Another possibility is a younger age for the not so well constrained Bajo de La Carpa Formation. Pissarrachampsinae also spans at least two depositional basins, whereas Baurusuchinae are endemic to a single basin.

Baurusuchia may be one of the most geographically and stratigraphically restricted radiations of crocodyliforms, but they have a relatively large morphological gap in relation to their closest sister taxa. Further discoveries are expected to fill in this gap and may expand the temporal and spacial range of the lineage. The only comparable crocodyliform clade, in terms of morphological distinctiveness and chronological/geographical restriction, is Mahajangasuchidae. The extreme morphological divergences of these taxa, compared to their sister clades, may in fact be partially explained by their temporal and spatial restrictions, similar to some extant isolated clades today.

### Conclusions

*Pissarrachampsa sera* is a distinct new baurusuchid from the Vale do Rio do Peixe Formation. The species' phylogenetic position was tested under a new morphological data set focusing on the differentiated morphology of the diagnostic members of the group. The data support a monophyletic Baurusuchidae and an internal split into Pissarrachampsinae, composed of *Wargosuchus* from Argentina and *Pissarrachampsa* described here, and Baurusuchinae composed of *Stratiotosuchus* and the three species of *Baurusuchus*. The morphological data support a big morphological gap between Baurusuchidae and its outgroups. Additionally, the affinity between *Wargosuchus* and *Pissarrachampsa* suggest an older age (possibly Santonian) for the Vale do Rio do Peixe Formation in Minas Gerais State.

## Supporting Information

Text S1 **Revised diagnosis of baurusuchid species of the ingroup included in the analyses**. The specimens circumscribed in each proposed diagnosis are listed. The references for the unique characters previously proposed are given *Cynodontosuchus rothi* [94], *Baurusuchus pachecoi* [94], *Stratiotosuchus maxhechti* [80], *Baurusuchus salgadoensis* [74] and *Baurusuchus albertoi* [22]. The referred material of *Stratiotosuchus maxhechti* was described by [80], but see Text S1.
(DOC)

Text S2 **OTUS used in Parsimony Analysis.** Specimens studied firsthand are listed. Descriptive accounts taken for the scoring of the OTUS are following *Notosuchus terrestris* [29], [83], [108], *Mariliasuchus amarali* [103], [109], *Armadillosuchus arrudae* [59], *Baurusuchus pachecoi* [1], [18], [22], [110], *Stratiotosuchus maxhechti* [62], [80], *Baurusuchus salgadoensis* [18], [111], *Wargosuchus australis* [21] and *Baurusuchus albertoi* [22], [112].
(DOC)

Text S3 **List of characters used in phylogenetic analysis.** The 66 characters used in the phylogenetic analyses are described (along with the character-states). The character–taxon matrix is presented in Text S4. Characters are either new or adapted from previously published studies. Character 1 was adapted from [4] (character 9), [11](character 65), [30] (character 103), [34] (character: 9); character 2 was adapted from [112] (character 257); character 7 was adapted from [4] (character 23), [113] (character 81); character 8 was modified from [4] (character 20); character 9 was adapted from[75] (Character 260); character 11 was adapted from [4] (character 23); character 12 was modified from [79](character247); character 14 was adapted from [115] (character 6), [4] (characters 19), [114] (character 80); character 16 was adapted from [74] (Character 258); character 17 was adapted from [6] (character 157); character 18 was adapted from [4] (characters 35 and 36) and [114] (character 140); character 19 was adapted from [4] (character 36); character 21 was adapted from [55] (character 181); character 22 was adapted from [4] (character 79) and [30] (character 107); character 24 was adapted from [81] (character 144); character 25 was adapted from [81] (character 144); character 26 was adapted from [114] (character 139); character 27 was adapted from [81] (character 133), [6] (character 145), [12] (character 121), [116] (character 46); character 28 was adapted from [4] (character 18); character 33 was adapted from [74]; character 38 was adapted from [30] (character 57); character 50 was adapted from [116] (character 103); character 54 was adapted from [55] (character 179); character 55 was modified from [27] (character 11), [30] (character 114); character 56 was adapted from [117] (character 27), [6] (character 133); character 57 was adapted from [4] (character 78); character 58 was adapted from [4] (character 78); character 59 was adapted from [116] (character 30); character 61 was adapted from [6] (character 90); character 62 was adapted from [30] (character 178); character 63 was adapted from [56] (character 181); character 65 was aAdapted from [4] (character 76), [30] (character 201); character 66 was adapted from[74].
(DOC)

Text S4 **Character-State Matrix.**
(DOC)

Text S5 **Synapomorphy List.**
(DOC)
