# Supplementary material for: A New Baurusuchid (Crocodyliformes, Mesoeucrocodylia) from the Late Cretaceous of Brazil and the Phylogeny of Baurusuchidae
Source: PLoS One. 2011 Jul 13;6(7):e21916. doi: 10.1371/journal.pone.0021916 (PMC3135595; doi:10.1371/journal.pone.0021916)
Supplement: Text S1 — Revised diagnosis of baurusuchid species of the ingroup included in the analyses. The specimens circumscribed in each proposed diagnosis are listed. The references for the unique characters previously proposed are given Cynodontosuchus rothi [94], Baurusuchus pachecoi [94], Stratiotosuchus maxhechti [80], Baurusuchus salgadoensis [74] and Baurusuchus albertoi [22]. The referred material of Stratiotosuchus maxhechti was described by [80], but see Text S1. (DOC) [file pone.0021916.s001.doc]

**Text S1.**

Characters previously proposed as unique for certain taxa are marked with an asterisk.

***Cynodontosuchus rothi* Woodward, 1896** . Museo de La Plata (MLP) 64‑IV‑16-25 - Baurusuchid with notch at the premaxilla-maxilla contact for the reception of enIarged anterior dentary tooth anteroposteriorly enlarged but dorsoventrally low*, palatine bar flat with wide ventral face, absence of row of foramina flanking the medial contact of palatines, absence of enlarged premaxillary teeth.

***Baurusuchus pachecoi* Price, 1945**. Museu de Ciências da Terra, Departamento Nacional de Produção Mineral (DGM) 299-R - Baurusuchid with lateral margins of squamosal and postorbital parallel to skull longitudinal axis, alveolar margin of the maxilla straight (in lateral view) posterior to the enlarged caniniform tooth, palatine bar with cylindrical dorsal portion and constricted posteriorly, maxilla-palatine suture V-shaped*, choanal septum transversally broad, ventral ridges of the basisphenoid sub-parallel.

***Stratiotosuchus maxhechti* Campos, Suarez, Riff & Kellner, 2001**. DGM 1477-R - Baurusuchid with three premaxillary teeth, ventral notch in the posterior portion of the jugal orbital border, anterior and posterior palpebrals sutured without a foramen between them, frontal does not enter the supratemporal fossa*, enlarged foramen incisivum in premaxilla-maxilla suture. A referred partial skull is reported for *Stratiotosuchus* Universidade Estadual Paulista, Rio Claro R 73 from Monte Alto, São Paulo, Brazil. This specimen presents some distinct morphological characters from the holotype, which were attributed to taphonomic distortions and ontogenetic variation. We have not analyzed this specimen firsthand; however, we point out some differences between this specimen and the holotype (e.g., the presence of a foramen between the palpebrals, presence of a small posttemporal fenestra and a non-ridged choanal septum) which vary among the phylogeny of Baurusuchidae proposed here. In addition, the referred specimen was collected more than 300 km from the holotype. This distance is bigger than any geographic range associated with undisputed Brazilian baurusuchids. Based on these concerns, we prefer to maintain only the holotype in our diagnosis and for OTU scoring.

***Baurusuchus salgadoensis* Carvalho, Arruda-Campos & Nobre, 2005**. Museu Histórico e Paleontológico de Monte Alto (MPMA)-62-0001-02 - Baurusuchid with paired ventral ridges of basisphenoid anteroventrally convergent and ridged border of the angular's medial surface does not overcome the anterior edge of the mandibular fenestrae*.

***Wargosuchus australis* Martinelli & Pais, 2008***.* Museo Professor J. Olsacher -PV 6134 - Baurusuchid with frontal-nasal contact, lacrimal duct placed ventrally to the corner formed by the dorsal and lateral lacrimal surfaces, dorsal surface of the anterior palpebral raised in relation to that of the prefrontal with a groove between those surfaces.

***Baurusuchus albertoi* Nascimento & Zaher 2010 -** Museu de Zoologia da Universidade de São Paulo -PV 140 – Baurusuchid with the descending expansion of the lateral edge of the squamosal anteriorly concave, well-developed ventromedial crest on the quadrate*.
