# Supplementary material for: A New Baurusuchid (Crocodyliformes, Mesoeucrocodylia) from the Late Cretaceous of Brazil and the Phylogeny of Baurusuchidae
Source: PLoS One. 2011 Jul 13;6(7):e21916. doi: 10.1371/journal.pone.0021916 (PMC3135595; doi:10.1371/journal.pone.0021916)
Supplement: Text S2 — OTUS used in Parsimony Analysis. Specimens studied firsthand are listed. Descriptive accounts taken for the scoring of the OTUS are following Notosuchus terrestris [29], [83], [108], Mariliasuchus amarali [103], [109], Armadillosuchus arrudae [59], Baurusuchus pachecoi [1], [18], [22], [110], Stratiotosuchus maxhechti [62], [80], Baurusuchus salgadoensis [18], [111], Wargosuchus australis [21] and Baurusuchus albertoi [22], [112]. (DOC) [file pone.0021916.s002.doc]

**Text S2.**

The OTUs used in the parsimony are listed below, along with data sources for the phylogenetic analysis. *Pissarrachampsa sera* was coded based on both LPRP/USP 0019 and LPRP/USP 0018. Holotypes and lectotypes are marked with an asterisk.

**Outgroup**

***Notosuchus terrestris* Woodward, 1896 -** Museo de Geología y Paleontología, Universidad Nacional del Comahue (MUCP) 137, MUCP 147, Museo Argentino de Ciencias Naturales (MACN)-PV-RN-1037; MACN-PV-RN-1038; MACN-PV-RN-1045, MLP-64-IV-16-5*.

***Mariliasuchus amarali* Carvalho & Bertini, 1999 –** Coleção de Paleontologia de Vertebrados da Universidade Federal do Rio de Janeiro (UFRJ) DG 50R*, UFRJ DG 105R, UFRJ DG 106R.

***Armadillosuchus arrudae* Marinho & Carvalho, 2009 -** UFRJ DG 303R*, MPMA-64-0001-04.

**Ingroup**

***Cynodontosuchus rothi* Woodward, 1896 -** MLP 64‑IV‑16-25*.

***Baurusuchus pachecoi* Price, 1945 –**This taxon was scored only based on published descriptive accounts.

***Stratiotosuchus maxhechti* Campos, Suarez, Riff & Kellner, 2001 -** DGM 1477-R*.

***Baurusuchus salgadoensis* Carvalho, Arruda-Campos & Nobre, 2005 –** MPMA-62-0001-02*.

***Wargosuchus australis* Martinelli & Pais, 2008 -** Museo Professor J. Olsacher -PV 6134**.*

***Baurusuchus albertoi* Nascimento & Zaher 2010 -** This taxon was scored only based on published descriptive accounts.
