# Supplementary material for: A New Baurusuchid (Crocodyliformes, Mesoeucrocodylia) from the Late Cretaceous of Brazil and the Phylogeny of Baurusuchidae
Source: PLoS One. 2011 Jul 13;6(7):e21916. doi: 10.1371/journal.pone.0021916 (PMC3135595; doi:10.1371/journal.pone.0021916)
Supplement: Text S3 — List of characters used in phylogenetic analysis. The 66 characters used in the phylogenetic analyses are described (along with the character-states). The character–taxon matrix is presented in Text S4. Characters are either new or adapted from previously published studies. Character 1 was adapted from [4] (character 9), [11](character 65), [30] (character 103), [34] (character: 9); character 2 was adapted from [112] (character 257); character 7 was adapted from [4] (character 23), [113] (character 81); character 8 was modified from [4] (character 20); character 9 was adapted from[75] (Character 260); character 11 was adapted from [4] (character 23); character 12 was modified from [79](character247); character 14 was adapted from [115] (character 6), [4] (characters 19), [114] (character 80); character 16 was adapted from [74] (Character 258); character 17 was adapted from [6] (character 157); character 18 was adapted from [4] (characters 35 and 36) and [114] (character 140); character 19 was adapted from [4] (character 36); character 21 was adapted from [55] (character 181); character 22 was adapted from [4] (character 79) and [30] (character 107); character 24 was adapted from [81] (character 144); character 25 was adapted from [81] (character 144); character 26 was adapted from [114] (character 139); character 27 was adapted from [81] (character 133), [6] (character 145), [12] (character 121), [116] (character 46); character 28 was adapted from [4] (character 18); character 33 was adapted from [74]; character 38 was adapted from [30] (character 57); character 50 was adapted from [116] (character 103); character 54 was adapted from [55] (character 179); character 55 was modified from [27] (character 11), [30] (character 114); character 56 was adapted from [117] (character 27), [6] (character 133); character 57 was adapted from [4] (character 78); character 58 was adapted from [4] (character 78); character 59 was adapted from [116] (character 30); character 61 was [file pone.0021916.s003.doc]

**Text S3.**

Characters 5, 11, 12, 27 and 32 were treated as additive and are marked with a +, autapomorphies are marked with an asterisk.

1. Premaxilla-maxilla suture: in the lateral surface of rostrum (0), internalized in a notch for the reception of lower caniniform (1).
2. Nasals: paired (0), partially or complete fused (1).
3. Posterior portion of the nasal elevated above the dorsal surface of the maxillae, forming a sagittal bar: absent (0); present posteriorly (1). New character. Crocodyliformes in general have a smooth transition between the dorsal surface of the nasal and maxilla at the posterior portion of the rostrum, as found in *Mariliasuchus*, *Hamadasuchus*, and *Crocodylus*. However, within Baurusuchidae, the posterior portion of the nasal is markedly elevated in relation to the dorsal surface of the maxilla, forming an acute bar which tends to become less marked anteriorly, becoming confluent with the dorsal surface of the maxillae just anterior to the orbit.
4. Dorsal surface of the nasal, posterior portion: rounded or flat (0), bearing a rugose broad depression (1). New character. Usually in Crocodyliformes, the posterior portion of the nasals has a central portion that is at the same level, or above, the borders of the bone, as seen in *Baurusuchus*. On the other hand, *Pissarrachampsa* and *Wargosuchus* have the lateral borders of the posterior portion of the nasal elevated in relation to the central portion of the bone. In addition, the dorsal surface of their rostrum has a ventrally directed dorsal curvature. The combination of both characters with the highly rugose bone surface in this region forms an extensive rugose depression typical to those taxa.
5. Prefrontal-prefrontal medial contact (+): absent (with a broad contact between nasal and frontal) (0) medial margin of prefrontals anteriorly convergent, almost touching each other (with a tiny contact between nasal and frontal) or touching each other anteriorly (1), contact present along mostly of the dorsal medial edge (2). New character. Crocodyliformes in general have a broad contact between frontal and nasal (e.g., *Mariliasuchus*, *Hamadasuchus*, *Sebecus*, *Crocodylus*) preventing contact between the contralateral prefrontals, but baurusuchids have a median approximation of the prefrontal. In *Pissarrachampsa* and *Wargosuchus* the prefrontal approximation occurs only at the anterior portion of the bone, whereas the posterior region remains well separated. In *Stratiotosuchus* and *Baurusuchus* the prefrontals contact along mostly their medial margins.
6. *Dorsal surfaces of prefrontal and anterior palpebral: continuous (0), central portion of the dorsal surfaces elevated, with a marked groove between them (1). New character. In *Wargosuchus* the central portions of the dorsal surfaces of the palpebral and nasal are raised, forming a groove between the elements. In other baurusuchids (e.g., *Pissarrachampsa*, *Stratiotosuchus*) the central part of those bones are about the same level as the lateral and medial portions of the dorsal surface.
7. Frontal: participates in the supratemporal fenestrae (0), excluded from the supratemporal fenestrae (1).
8. Width of frontals between orbits relative to mid-length width across nasals: narrow (similar to width of nasals) (0); or broad (about twice the width of nasals) (1).
9. Dorsal surface of frontal, posterior to orbits: flat or slightly concave (0), or markedly concave transversely (1).
10. Midline longitudinal depression on anterior portion of frontal: absent (0), present (1). New character. The general condition of the frontal anterior surface is flat to rounded, as seen in *Mariliasuchus* and *Baurusuchus pachecoi*. *Pissarrachampsa* and *Wargosuchus* show a modified condition in which a conspicuous depression occurs along the midline of this region. A similar condition is also found in *Calsoyasuchus valliceps* and was previously termed the ‘frontal dorsal valley’.
11. Frontal longitudinal ridge (+): absent (0), restricted to the posterior portion (1), extending anteriorly to the frontal mid-length (2).
12. Support for the anterior palpebral bone (+): marked depression on prefrontal (0), marked depression on prefrontal and lacrimal forming an incipient lateral projection (1), marked depression on prefrontal and lacrimal forming a great lateral projection for the support of anterior palpebral (2)..
13. *Supratemporal rims raised and hypertrophied: restricted to the median edge of the external supratemporal fenestra (0), extended to anterior edge of the external supratemporal fenestrae (1). New character. In baurusuchids, as seen in *Stratiotosuchus*, the median borders of the external supratemporal fenestrae are raised and hypertrophied, forming a rim. In *Pissarrachampsa* that rim is not limited to the medial border, but also extends anteriorly, following the lateral curvature of the anterior border of these openings and almost reaching the lateral contact between frontal and posterior palpebral.
14. Postorbital-quadratojugal contact in lateral view: restricted (0), broad contact between quadratojugal and the posterior portion of the postorbital descending flange (1).
15. Lateral surface of the postorbital descending flange: flat (0), or concave (1). New character. The general condition of Crocodyliformes is a restricted postorbital descending flange, as seen in *Crocodylus*, or a flat well developed postorbital descending flange, as in *Mariliasuchus*. In baurusuchids, the well developed descending flange of the postorbital is markedly concave, as seen in *Pissarrachampsa*.
16. Postorbital-squamosal suture at the skull table in lateral view: straight line (0), squamosal anteriorly convex (1).
17. Lateral margins of squamosal and postorbital in dorsal view: parallel (0), or diverging posteriorly (1).
18. Squamosal prongs: present (0), absent (1).
19. Squamosal lateral descending flange: obliquely directed (0), vertically directed with lateral edge medially concave and laterally convex (1), vertically directed and medially convex and laterally concave (2).
20. *Lacrimal duct external aperture: ventral to the corner formed by the dorsal and lateral lacrimal surfaces (0), at the corner (1). New character. The participation of the lacrimal in the structure that supports the anterior palpebral, as seen in Baurusuchidae(e.g., *Wargosuchus*), defines two surfaces of this bone. The lateral surface is continuous to the lateral surface of the rostrum, whereas the dorsal surface is perpendicular to the former, facing dorsally, and contacts the prefrontal lateral expansion. In *Pissarrachampsa*, the lacrimal duct opening is located on the lateral corner formed by those two surfaces, while in other taxa (e.g., *Wargosuchus*) the opening is located above the corner formed by the two planes.
21. *Contact between anterior and posterior palpebrals: only laterally, forming a supraorbital foramen (0), along the whole length without a foramen (1).
22. Alveolar margin of maxilla in lateral view: almost straight (0), arched only anteriorly, below the enlarged caniniform tooth (1), arched along the entire alveolar margin (2).
23. Maxillary palatal sagittal contact: smooth (0), bearing a longitudinal series of foramina (1). New character. In general the maxillae palatal surface is flat and smooth (e.g., *Crocodylus*) or bears a longitudinal *torus* (e.g., *Hamadasuchus*). Derived Baurusuchidae show a modified condition, in which a series of longitudinal irregularly spaced foramina are placed at the median palatal contact of the maxillae, so that the foramina are bounded by both bones of the pair.
24. Dorsoventral depth of the jugal orbital portion in relation to infratemporal portion: almost the same depth (0), orbital portion twice the depth of the infratemporal portion (1).
25. Dorsoventral depth of the jugal antorbital portion in relation to infraorbital portion: equal or lower (0), antorbital portion deeper than infraorbital portion (1).
26. *Posterior portion of the jugal orbital border: without a notch (0), with a marked ventral notch (1).
27. Jugal outer surface (+): confluent along the entire length (0), infratemporal portion of jugal laterally displaced but not reaching the level of orbital anterior edge (1), infratemporal portion of jugal laterally displaced and reaching the level of orbital anterior edge (2).
28. Jugal infratemporal bar: laterally flat (0), rod-shaped (1).
29. Anterodorsal ramus of quadrate in ventral view: developed, forming more than 50% of the lateral edge of the internal supratemporal fenestra (0), restricted, forming less than 50% of the lateral edge of the internal supratemporal fenestra (1). New character. In general the quadrate of Crocodyliformes emits a dorsal process extending medial to the postorbital descending flange and the quadratojugal dorsal process, which forms most of the lateral edge of the internal supratemporal fenestrae (e.g., *Alligator*). In Baurusuchidae such as *Pissarrachampsa* this process is less extensive anteriorly and does not medially cover the postorbital and quadratojugal. Accordingly, the postorbital and quadratojugal form most of the lateral edge of that opening.
30. *Ventral border of quadratojugal and quadrate (lateral view): continuous (0), quadratojugal ventrally displaced, forming a notch between the bones (1). New character. Crocodyliformes usually posses a continuous ventral margin of the temporal area, formed by the quadratojugal and jugal (**e.g.;** *Mariliasuchus*, *Baurusuchus pachecoi*). In *Pissarrachampsa*, on the other hand, the ventral margin of the quadratojugal is more ventrally expanded, so that a notch is formed just posterior to the quadratojugal where it meets the quadrate.
31. Anterior margin of distal portion of body of the quadrate: at a right angle to the anterolateral portion of the bone (0), slopes gently towards the anterolateral portion of the bone (1). New character. In Crocodyliformes with a verticalized distal body of the quadrate, the transition between this portion of the bone and its anterolateral portion is abrupt, forming an almost right angle, as in *Armadillosuchus* and *Mariliasuchus*. In Baurusuchidae (e.g., *Pissarrachampsa*), the anterior face of the quadrate distal body slopes gradually to the anterior portion of the bone, forming a smooth transition between these two regions of quadrate.
32. Quadratojugal dorsal ramus in medial view ( +): ending ventrally, or at the same level, of the dorsal tip of laterotemporal fenestra (0), overcoming the dorsal tip of laterotemporal fenestra but not reaching the supratemporal fossa (1), overcoming the dorsal tip of laterotemporal fenestra and reaching the supratemporal fossa (2). New character. The quadratojugal of Crocodyliformes possesses a dorsally directed ramus that overlaps the quadrate medially and borders the infratemporal fenestrae. The dorsal extension of this ramus varies among the studied taxa and does not symmetrically matches the lateral exposure of the bone. In Baurusuchinae (**e.g.;** *Stratiotosuchus*), the medial face does not overcomes the dorsal tip of the infratemporal fenestrae, whereas in the other taxa the medial face overcomes that dorsal tip (as seen in *Armadillosuchus*). In *Pissarrachampsa*, this condition is exaggerated so that the medial face reaches the ventral margin of the supratemporal fossa.
33. Quadrate lateral depression: absent (0), present with the major axis dorsoventrally oriented (1), present with the major axis anteroposteriorly oriented and with quadratojugal-quadrate suture within the depression (2).
34. Quadrate fenestrae: visible in lateral view (0), internalized in otic notch (1). New character. Additional quadrate fenestrae are commonly found in Crocodyliformes, arranged around the otic aperture, as seen in the protosuchian *Zosuchus davidsoni* and in *Pissarrachampsa*. In Baurusuchinae (e.g., *Stratiotosuchus*) the margin of the otic notch is more developed, encompassing the region of the additional quadrate fenestrae and hiding those apertures so that only the anterior one is partially visible in lateral view.
35. Lateral quadrate condyle: almost as anteroposteriorly wide as the medial condyle (0), or lateral quadrate condyle hemispherical (1). New character. In general, the anteroposterior extension of the medial and lateral quadrate condyles are subequal, as seen in *Notosuchus.* However, in some of the analyzed taxa (e.g.: *Baurusuchus salgadoensis*) the lateral condyle is enlarged in relation to the medial condyle, and has a hemispherical appearance in ventral view.
36. Muscle scar in the medial quadrate surface (ridge ‘A’): curved or almost straight (0), sigmoidal (1). New character. The muscle scar located at the ventromedial surface of the quadrate, associated to ‘ridge A’ of eusuchians, possesses a straight to curved profile in basal Mesoeucrocodylia, as in *Baurusuchus salgadoensis*. In some of the studied taxa (e.g., *Pissarrachampsa*) this structure assumes an evident sigmoidal profile, with the anteroventral orientation of its main axis.
37. Supraoccipital dorsal exposure: along the midline portion of posterior region of skull table (0), restricted to a thin surface attached to the posteriormost portion of parietal and squamosal (1). New character. The supraoccipital dorsal exposure on the skull roof varies in different Crocodyliformes groups, ranging from its total absence (e.g., *Hamadasuchus*) to a great median dorsal extension (e.g., *Notosuchus*). In Baurusuchidae (e.g., *Pissarrachampsa sera* and *Baurusuchus salgadoensis*), the supraoccipital assumes a unique morphology, and has a restricted thin transversal exposure in the posteriormost region of the skull roof. This exposure possesses the same anteroposterior thickness for its whole length until the inflexion point of the bone towards the occipital plane of the skull.
38. Posterior region of the auditory fossa: posteriorly open (0), bound posteriorly by a posteroventrolateral extension of the squamosal and exoccipital (1).
39. Foramen incisivum: absent or small (0), enlarged (1). New character. The presence, as well the relative size of the incisivum foramen in the premaxilla-maxilla suture varies greatly among Crocodyliformes. Within the analyzed taxa, this structure is absent ( e.g., *Mariliasuchus*) or relictual (e.g., *Baurusuchus pachecoi*) in most taxa, but well developed in *Cynodontosuchus* and *Stratiotosuchus*.
40. Anterior extension of palatine: passes the anterior margin of the suborbital fenestrae (0), does not reach the level of the anterior margin of suborbital fenestrae (1). New character. Crocodyliformes in general has the palatine extending anterior to the anterior border of the suborbital fenestra. This extension can have different shapes, ranging from just a small anterior projection (e.g., *Alligator*) to a much broader structure, so that the palatine borders most of anterior margin of this opening (e.g., *Hamadasuchus*). In Baurusuchidae the palatine-maxilla suture is located posterior to the anterior margin of the suborbital fenestrae (e.g., *Cynodontosuchus*, *Pissarrachampsa*, *Baurusuchus pachecoi*), and the palatine does not contribute to the anterior margin of this aperture.
41. Ventral face of palatine bar: flat and wide (0), ventral surface restricted and dorsal portion cylindrical with the same wideness through (1) ventral surface restricted and dorsal portion cylindrical constricted in the posterior portion (2). New character. Usually for Mesoeucrocodylia the palatine bar has a large flat ventral surface that does not allow the observation of the dorsal portion of the bone in ventral view as seen in eusuchians (e.g., *Alligator*) and *Cynodontosuchus*. On the other hand, in advanced baurusuchids, the ventral surface of palatine is restricted to a narrow surface, and the dorsal cylindrical portion of the bone is ventrally apparent (e.g., *Pissarrachampsa*, *Baurusuchus pachecoi*). Furthermore, the dorsal cylindrical surface can have the same wideness through (e.g., *Pissarrachampsa*) or be constricted at its posterior portion, near the palatine posterolateral divergent rami (e.g., *Baurusuchus salgadoensis*).
42. Medial palatal contact: smooth (0), rugose (1). New character. The medial contact of the palatines in Crocodyliformes is usually flat (e.g., *Cynodontosuchus*, *Lomasuchus*) or often somewhat sulcate (e.g., *Alligator* *mississipiensis*), but in Baurusuchia that contact is distinctly raised, forming a ridged rugose suture along its whole extension.
43. *Dorso-lateral surface of the ectopterygoid near jugal contact: smooth (0), bearing a series of irregularly-sized foramina (1). New character. The ectopterygoid of most analyzed taxa has a robust constitution, with a large and generally smooth lateral surface that curves dorsolaterally to meet the jugal (e.g., *Mariliasuchus*, *Baurusuchus albertoi*). *Pissarrachampsa*, on the other hand, shows a series of irregularly-sized foramina at this broad laterally exposed surface.
44. Row of foramina flanking the medial contact of the palatines: absent (0), present (1). New character. The palatine bar of Crocodyliformes usually has a smooth ventral surface (e.g., *Mariliasuchus*, *Cynodontosuchus*). The restricted ventral surface of that bar in some baurusuchids bears a series of foramina located in a pair shallow grooves flanking the median contact of the bones (e.g., *Pissarrachampsa sera*, *Baurusuchus pachecoi*). These foramina are regularly spaced and follow, for a short distance, the lateral curvature of the palatal posteroventral divergent rami where both grooves become confluent.
45. Ridge on the ectopterygoid-jugal articulation: absent (0), present and continuous with the ventral ridge of the infratemporal portion of jugal (1), present but separated from the ventral ridge of the infratemporal portion of jugal by a notch at the posterior margin of the articulation (2). New character. Usually for Crocodyliformes the jugal-ectopterygoid contact is flat and located in the ventral surface of the skull (e.g., *Hamadasuchus*, *Mariliasuchus*). Baurusuchids posses the jugal-ectopterygoid suture located laterally in the skull. In addition, the contact between these bones is markedly ridged, partially because of the dorsolateral curvature of the ectopterygoid, and partially due to the more robust ventral margin of the jugal. This ridged suture extends along the ventral portion of cheek area and is either continuous with the laterally displaced ridge of the infratemporal ramus of the jugal (e.g., *Baurusuchus pachecoi*) or separated (e.g., *Pissarrachampsa*) by a notch at the posteriormost region of the ectopterygoid-jugal suture.
46. *Ectopterygoid-jugal contact behind pterygoid wing in ventral view: rounded (0), medially angled (1). New character. The ectopterygoid body in derived Notosuchia, including Baurusuchidae, is a anteromedially oriented, robust element. Its posterior end is rounded in most taxa (e.g., *Stratiotosuchus*), but in *Pissarrachampsa*, the posterior portion of the ectopterygoid-jugal suture forms a square angle between a medially directing segment perpendicular to the jugal main axis.
47. Ventral surface of the choanal septum: smooth (0), ridged (1). New character. The choanal septum of most of the analyzed taxa has a flat to rounded ventral surface, which is the variation found in most crocodyliforms (e.g., *Mariliasuchus*, *Pissarrachampsa sera*, *Sebecus querejazus*). In *B. pachecoi* and *Stratiotosuchus*, on the other hand, the central portion of the septum is markedly ridged and ventrally displaced relative to the adjacent regions.
48. Major surface of the pterygoid wing: lateroventrally oriented (0), ventrally oriented (1). New character. The pterygoid wing of Crocodyliformes is usually a flat structure with its main surface ventrally directed (e.g., *Mariliasuchus*, *Pissarrachampsa*, *Alligator mississipiensis*). However, in the species of *Baurusuchus*, the pterygoid wing is anteriorly twisted, so that the main surface is anterolaterally directed. This peculiar orientation makes the ectopterygoid as anteroposteriorly long as the pterygoid.
49. Posttemporal fenestrae: present (0), absent (1). New character. The size and location of the posttemporal fenestrae in Crocodyliformes varies across different group, probably with multiples gain/loss events. Within the analyzed taxa, it is either absent (e.g., *Notosuchus*, *Pissarrachampsa*) or found as a small opening (e.g., *Mariliasuchus amarali*, *Baurusuchus pachecoi*).
50. Pterygoid parachoanal fenestra and depressions: absent (0), present (1).
51. Basisphenoid ventral surface: continuous to surrounding bones (0), ventrally displaced and separated from the neighboring elements by a posteroventral step formed by a groove that separated the bone from the main occipital plane (forming a postchoanal pterygoid-basisphenoid tuberosity) (1). New character. The basisphenoid ventral exposure varies greatly within Crocodyliformes. In the analyzed taxa, as well as in other basal Mesoeucrocodylia, the basisphenoid is largely exposed ventrally. In the outgroup taxa (e.g., *Armadillosuchus*), the ventral surface of the bone is confluent with the other neurocranial bones, whereas in Baurusuchidae (e.g., *Pissarrachampsa sera*, *Baurusuchus salgadoensis*) its central portion is ventrally projected, forming a step between this surface and that of the surrounding bones. Because of this ventral projection, the basisphenoid is delimited by a tuberosity that is especially developed anteriorly and termed here as the postchoanal pterygoid-basisphenoid tuberosity.
52. Anterior wall of the lateral Eustachian foramina: present, separating each foramen from the sulcus (0), absent, foramina open into the sulcus (1). New character. The lateral Eustachian foramina are usually bordered anteriorly by a wall formed by the basisphenoid alone (e.g., *Mariliasuchus*), or by the suture of that bone with the pterygoid (e.g., *Araripesuchus wegneri*, *Montealtosuchus*). In some of the analyzed taxa (e.g., *Armadillosuchus*, *Pissarrachampsa sera*, *Baurusuchus salgadoensis*) this anterior wall is absent and the aperture of each lateral Eustachian foramina faces anterolaterally instead of strictly ventrally.
53. Lateral Eustachian foramen: smaller than medial one (0), as large or larger than medial one (1). New character. In most Crocodyliformes the median Eustachian foramen is larger than the lateral elements (e.g., *Alligator*), as also seen in part of the analyzed outgroup (e.g., *Notosuchus*) and ingroup (e.g., *Baurusuchus salgadoensis*). On the other hand, *Armadillosuchus* and *Pissarrachampsa* posses enlarged lateral Eustachian foramina, which are more developed than the medial one.
54. Paired ridges located medially on the ventral surface of the basisphenoid (originating at the anterior margins of lateral Eustachian foramina): absent (0), present and anteroventrally convergent (1), present and sub-parallel (2).
55. Tooth mesial and distal carinae: smooth (0), bearing a pebbled surface (1), serrated (2).
56. Premaxillary teeth: four (0), three (1).
57. *Enlarged premaxillary teeth: present (0), absent (1).
58. Last premaxillary tooth hypertrophy: present (0), absent (1).
59. *Maxillary teeth: five or more (0), only four (1).
60. Mandibular outer surface sculpture: present on dentary (0), present on dentary and splenial (1). New character. Ornaments on the anterior mandibular outer surface are usually restricted to the dentary in Crocodyliformes (e.g., *Notosuchus*, *Alligator*), but in some of the analyzed taxa the ventral (outer) surface of the splenial is also sculptured (e.g., *Pissarrachampsa sera*, *Baurusuchus salgadoensis*).
61. Foramen intramandibularis oralis: small or absent (0), big and slot-like (1).
62. Mandibular symphysis, orientation of anterior part: horizontal or slightly dorsally directed (0), forming an angle of approximately 45 degrees to the main axis of the jaw (1).
63. Peg at the posterior surface of the mandibular symphysis formed by a ridged splenial suture: absent (0), present (1).
64. Posteroventral symphyseal depressions: absent (0), present (1). New character. The common condition found in basal Crocodyliformes is a vertically oriented splenial forming the posterior surface of the jaw symphysis (e.g., *Notosuchus*, *Alligator mississipiensis*). Yet, in various Mesoeucrocodylia the splenial enters ventral surface of the jaw (e. g., *Montealtosuchus*), but only in Baurusuchidae this area bears two distinct depressions (e.g., *Cynodontosuchus,* *Pissarrachampsa*, *Baurusuchus salgadoensis*). One depression is located at the level of dentary-splenial suture, dorsally displacing the entire posteroventral surface of the splenial. A second depression is located at the posteroventral surface of the splenial, displacing the posterior half of this surface more dorsally.
65. *M. pterygoideous posterior* insertion: reaching the anterior external mandibular fenestra (0), in a marked depression on surangular-angular lateral surface on posterior edge of external mandibular fenestra (1).
66. * Ridged border of the angular medial face: overcomes the anterior edge of the mandibular fenestra (0), does not overcome the anterior edge of mandibular fenestra (1).
