# Supplementary material for: A New Baurusuchid (Crocodyliformes, Mesoeucrocodylia) from the Late Cretaceous of Brazil and the Phylogeny of Baurusuchidae
Source: PLoS One. 2011 Jul 13;6(7):e21916. doi: 10.1371/journal.pone.0021916 (PMC3135595; doi:10.1371/journal.pone.0021916)
Supplement: Text S4 — Character-State Matrix. (DOC) [file pone.0021916.s004.doc]

**Text S4.**

Character states (0-2) are given for outgroup and ingroup taxa. Brackets enclose variable conditions; question mark indicates missing data; and a dash indicates inapplicability due to extreme transformation.

*Notosuchus terrestris*

00000?1000 200000000- 1000-00000 0?000?0000 00?00?0110 0000000000 00000?

*Mariliasuchus amarali*

0000001000 01000{0 1}0000 1000-01000 01001-0000 0000000101 0000100100 001000

*Armadillosuchus arrudai*

0100000000 0100111000 -????01110 01001100?? ?????????? 01101????? ??1???

*Cynodontosuchus rothi*

1????????? ?????????? ?{1 2}???????? ????????11 00?0?????? ????211-0? ?111??

*Wargosuchus australis*

11?111???1 ?2???????0 ?????????? ?????????? ?????????? ????2?00?? ??????

*Pissarrachampsa sera*

1111100011 22111{0 1}11{1 2}1 1211002111 12200111?1 1111210111 1112210011 1111??

*Stratiotosuchus maxhechti*

1100201110 1201101110 0211012110 1011001111 1???201?1? ??0?210001 1?11??

*Baurusuchus albertoi*

?????????? ???111112? 1??11021?0 101110?1?? ??0?2??0?? ?????????? ????10

*Baurusuchus pachecoi*

11???????? ???11101{1 2}? ?1111021?0 101110?101 21?11?100? 11?220010? 111?10

*Baurusuchus salgadoensis*

1110200110 1201111110 12?1102110 1011101101 21011??00? 1101200101 111111
