# Supplementary material for: A New Baurusuchid (Crocodyliformes, Mesoeucrocodylia) from the Late Cretaceous of Brazil and the Phylogeny of Baurusuchidae
Source: PLoS One. 2011 Jul 13;6(7):e21916. doi: 10.1371/journal.pone.0021916 (PMC3135595; doi:10.1371/journal.pone.0021916)
Supplement: Text S5 — Synapomorphy List. (DOC) [file pone.0021916.s005.doc]

**Text S5.**

List of apomorphic states by node for the minimum-length tree in the maximum-parsimony analysis (Figure 17). Delayed transformations are indicated with an asterisk.

*Armadillosuchus* + Baurusuchidae

2 (1), 7(0), 15 (1), 17(1), 28(1), 29(1), 52 (1).

Baurusuchidae

1(1); 22(2); 40(1)*; 55(2); 56(1)*; 62(1)*; 64(1)*.

Pissarrachampsinae + Baurusuchinae

5(1)*, 9(1)*, 12(2)*, 14(1)*, 18(1)*, 19(1)*, 23(1)*, 24(1)*,27(2)*, 31(1)*, 33(1)*, 37(1)*, 38(1)*, 41(1), 42(1); 44(1), 45(1)*, 51(1)*, 54(1)*, 60(1)*, 61(1)*.

Pissarrachampsinae

4(1), 5(1), 10(1).

Baurusuchinae

5(2), 8(1), 11(1)*, 32(0), 33(1)*, 34(1), 36(0), 47(1).

*Baurusuchus*

25(1), 48(0)*, 65(1)*

*B. pachecoi* + *B. salgadoensis*

45(1), 41(2)*, 56(0)*, 58(1)
